# 3D anthropometry of the nasolabial region in children aged 3 to 9 months as reference database for clinical assessment

**DOI:** 10.1038/s41598-025-11024-8

**Published:** 2025-07-28

**Authors:** Manuel Olmos, Joy Backhaus, Manuel Weber, Ragai Matta, Christoph Vogl, Katja Schulz, Sandra Beyer, Linus Winter, Anne Schützenberger, Marco Kesting, Rainer Lutz

**Affiliations:** 1 Department of Oral and Cranio-Maxillofacial Surgery, Friedrich-Alexander-Universität Erlangen-Nürnberg, Glueckstrasse 11, 91054 Erlangen, Germany; 2 Friedrich-Alexander-Universität Erlangen-Nürnberg (FAU), Erlangen, Germany; 3 Institute of Medical Teaching and Medical Education Research, University Hospital of Würzburg, Würzburg, Germany; 4 Department of Prosthodontics, Friedrich-Alexander-Universität Erlangen-Nürnberg, Erlangen, Germany; 5 Division of Phoniatrics and Pediatric Audiology, Department of Otorhinolaryngology Head & Neck Surgery, Friedrich-Alexander-Universität Erlangen-Nürnberg, Erlangen University Hospital, Erlangen, Germany

**Keywords:** 3D, Anthropometry, Cleft surgery, Assessment, Reference, Alar base types, Evaluation, Anatomy, Oral anatomy, Outcomes research, Paediatric research, Oral diseases, Development

## Abstract

Treatment for a cleft lip can result in significant functional and aesthetic changes to the nasolabial region. Although three-dimensional (3D) measurements are the gold standard for evaluating cleft surgery, most short- and long-term evaluations still rely on subjective assessment or the measurement of patient photographs. To our knowledge, this work establishes the first baseline and reference group for the nasolabial region in children aged 3 to 9 months without cleft lip or palate. This group can be used for future evaluations, such as those of surgical outcomes or NAM therapy, via 3D anthropometric measurement. Data was collected cross-sectionally from 25 children aged 3 to 9 months using a validated intraoral scanner (Trios 4, 3Shape). Scans were analysed according to 3D anthropometric criteria by metrically accurate measurements of distances, surface curves and angles using 3D inspection software (GOM Inspect, Co. Zeiss, Jena, Germany). Results are presented as reference database combined with a step-by-step guide on the measurement methodology. For easy application all data are additionally presented in the form of formulae in which clinical data can be inserted. Based on the data from healthy children, we propose a new classification of alar base types ranging from 1 to 3. Unlike conventional assessment methods, surface curves and other 3D anthropometric tools provide a highly accurate and objective quantification of the anatomy of the nasolabial region and thus serve as a foundation for future clinical research on cleft lip surgery. Alar base type classification may influence future surgical approaches to cleft lip surgery.

**Supplementary Information:**

The online version contains supplementary material available at 10.1038/s41598-025-11024-8.

## Background

The treatment of congenital deformities of the nasolabial region has far-reaching consequences for the future lives of our young patients. It should therefore be the goal of every cleft surgeon to provide the best possible treatment in order to create both function and aesthetics. Today, long after Millard’s work, the field of cleft surgery continues to change and improve, or as Millard himself once stated: ‘Semper investigans, nunquam perficiens. Always searching, never achieving (perfection)’^1,2^.

In order to progress towards the optimal outcome, it is essential to visualise and define it. To date, many studies have assessed the ‘where have we got to?’ by evaluating the outcomes of cleft lip surgery using various aesthetic scoring systems or symmetry assessments^3–7^. So far, Loo et al. are one of the very few to assess the ‘where exactly do we want to get to’ by describing the anthropometic morphology of the ‘normal Cupid’s bow’ in healthy young children^8^.

In today’s era of three-dimensional (3D) analysis techniques, it is essential that we have reference databases of these ‘normal’ anthropometics combined with appropriate and precise evaluation methods. These tools are indispensable in order to identify the optimal, or in other words the most unobtrusive, result that we are looking for, and to analyse the aspects in which we have not yet achieved it. This will provide the basis for further improving surgeons’ understanding and skills, and therefore the surgical outcomes. By establishing an objective and valid basis for comparison, it will also allow us to evaluate the surgical outcomes in detail, and to improve the surgical techniques in a very targeted way. It is important to note that this is only possible if the databases’ range corresponds to the age at which primary surgical intervention takes place. Available literature suggests that this procedure takes place between three and nine months after birth^9,10^. The present work therefore focuses on this timeframe.

A recent shift towards 3D assessment in cleft surgery has led to the use of different data acquisition tools, ranging from 3D photography to the extraoral use of intraoral scanners^11–16^. As part of the validation of a suitable measurement tool, our group has recently demonstrated the superiority of the extraoral application of a commercially available intraoral scanner (3Shape, Trios4 and 5) in terms of accuracy and applicability in mapping the nasolabial region^12^. The use of this high-precision instrument, which is now well established, provides the opportunity to apply a novel measurement method using previously unidentified measurement parameters that differ from those used in conventional anthropometry.

The EUROCLEFT and SCANDCLEFT studies, with their respective scoring scales, have already established global standards for the assessment of outcomes following surgical correction of cleft lip and palate (CLP) defects^10,17^. Although both studies highlight a glaring lack of evidence in orofacial cleft surgery, they do not yet make use of new 3D anthropometry within the scope of the respective possibilities. The recent works of Bugaighis et al. and Xu et al. uses 3D instruments for image acquisition. However, they are limited to the conventional measurement parameters established in two-dimensional anthropometry, such as direct distances and angles^15,18^.

As we stand on the verge of transitioning from 2D to 3D assessment in cleft surgery, the aim of this study is to establish a baseline and reference group for future evaluations. Additionally, an accurate and reproducible methodology is to be established for the 3D assessment of the nasolabial region in children aged 3 to 9 months, taking into account surface reflections, surface curves and perpendicular lengths. As part of the study of healthy children, a classification of alar base types will be presented.

## Methods

### Patient acquisition

An intraoral scanner, previously established and validated by our working group^12^was used to acquire the nasolabial region of 47 healthy children aged 3 to 9 months, who presented to the Division of Phoniatrics and Paediatric Audiology at the Department of Otorhinolaryngology at Erlangen University Hospital. Three members of the working group performed the scans, and the scanner was calibrated before each process. Scans that were distorted or otherwise corrupted by the childrens’s movements were excluded, leaving 25 patients who met the requirements for 3D anthropometric measurement for further analysis. The average time taken was 3 min per child, and all children with nasolabial or generalised cranial deformities were excluded. Of the 25 children included, 13 were male and 12 were female. The mean age was 4 months and 18 days with a minimum age of 3 months and a maximum age of 8 months and 30 days. Birth weight was recorded as another demographic parameter.

All parents were informed in detail about the study and gave their informed written and verbal consent. All children’s legal guardians gave informed consent for the publication of identifying information/images in an online open access publication. The study was approved by the Ethics Committee of the Friedrich-Alexander University of Erlangen-Nürnberg on May 6, 2022 under the application number 22-100-B and all methods were performed in accordance with the relevant guidelines and regulations.

### Measuring methods

**Fig. 1:**
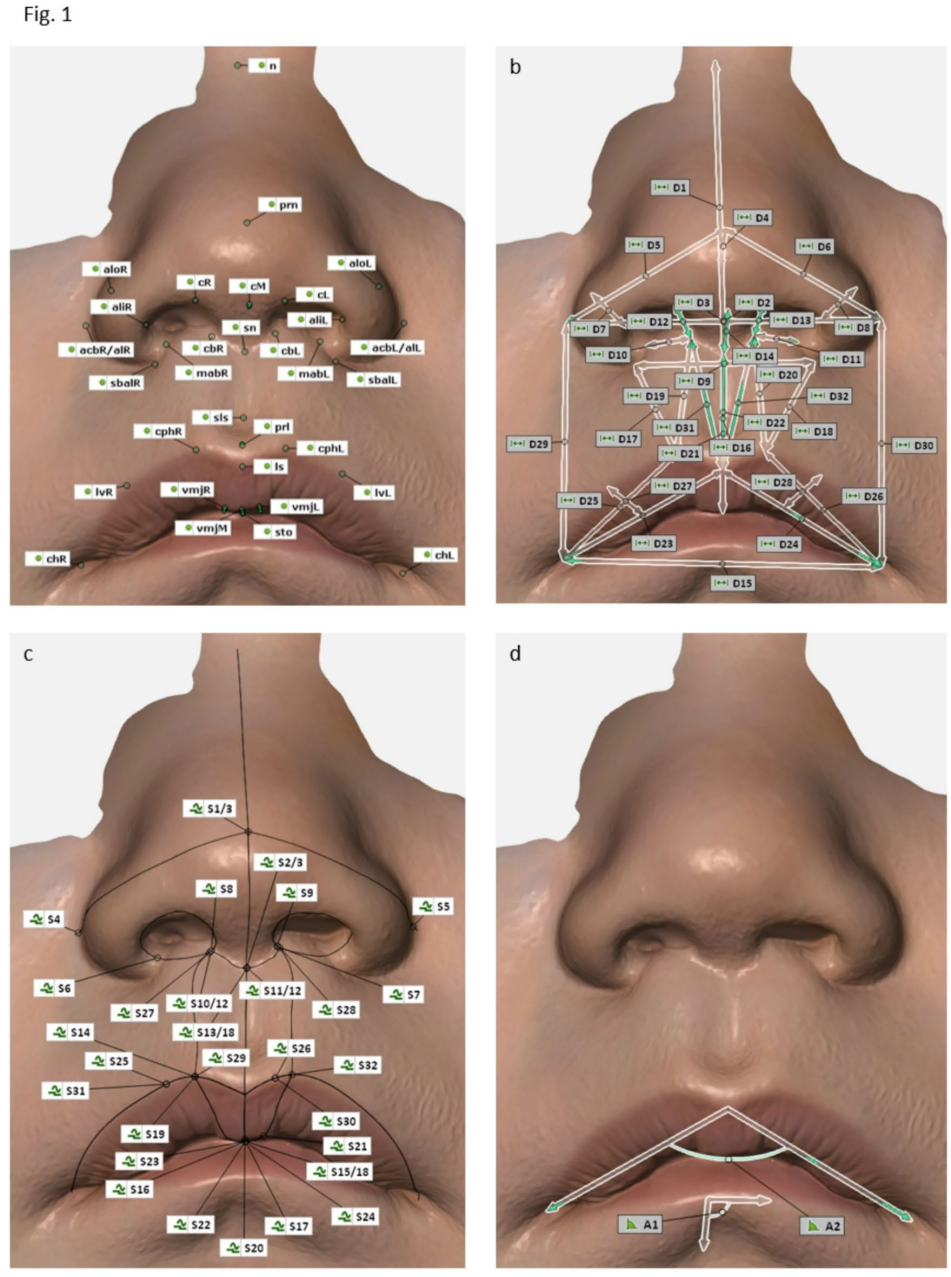
(**a**) Overview of the 3D anthropometric landmarks of the nasolabial region. The 33 landmarks are placed on the virtual surface, taking into account anatomical structures, colour/texture and surface reflection. Detailed instructions on placing the landmarks can be found in Supplementaray Material 1 and 4. Corresponding number assignments (e.g. L1 - Nasion) can be found in the table in Supplementary Material 1. (**b**) Overwiew of the 3D anthropometric distances of the nasolabial region The 32 distances are measured as a direct length between two landmarks or as a perpendicular length between a previous measurement and a new landmark, as in the example of effective nasal length, dimple depth or right and left vermillion convexity. Corresponding number assignments (e.g. D1 - Nasal height) can be found in the table in Supplementary Material 1. (**c**) Overwiew of the 3D anthropometric surface curves of the nasolabial region. The 32 surface curves are positioned directly on the mesh. They can be set by raw placement using landmarks only, or by using intersection points. Detailed instructions on placing the surface curves and corresponding number assignments (e.g. S1 - Volumetric upper nasal height) can be found in Supplementaray Material 1. (**d**) Overwiew ot the 3D anthropometric angles of the nasolabial region The 2 angles consist of a 3-point angle and a bipartite angle. The vermillion angle is constructed as a 3-point angle, the collumellar angle as a 2-directional angle. Corresponding number assignments (e.g. A1 - Columellar angle) can be found in the table in Supplementary Material 1.

The measurement methodology was derived from the seminal work of Leslie G. Farkas, namely ‘Anthropometry of the head and face’, as well as from the contributions from Chong et al., Bughaigis et al. and Kesting et al.^1,18–20^. Subsequently, our working group modified this methodology to take advantage of the new possibilities offered by modern 3D anthropometry. This involved focusing on light reflections and incorporating surface curves and perpendicular lengths. The basis of all our 3D anthropometric measurements were 33 landmarks (Fig. 1a; Table 1), i.e. points on a surface that can be defined as constructed elements without a vector. The step-by-step guide, which is presented in the results section, was developed with the aim of defining their precise location and facilitating their placement for future work. Our final reference database consists of 32 distances (Fig. 1b), 33 surface curves (Fig. 1c), 2 angles (Fig. 1d) and 18 indices (Table 2).

**Table 1:** Overview and definition of the 33 3D anthropometric landmarks of the nasolabial region. An additional table enhanced with images can be found in the supplementary material section (Supplementary Material 1).

Landmarks (abbreviation, timestamp for video tutorial)	Definition
Nasion (n; 0:02)	Point in the midline of the nasal root identical to the hard tissue nasion. 3D anthropometrically, the n point can be identified at the point of change in light reflection by repeatedly rotating the mesh around an imaginary transverse axis.
Subnasale (sn; 0:40)	Midpoint of maximum concavity where the upper lip skin meets the columella base. 3D anthropometrically, the landmark can be located based on the light reflection at the caudal columellar base on an imaginary line connecting prn and ls.
Pronasale (prn; 1:23)	The most prominent point of the nasal tip on the morphological median sagittal line. In 3D anthropometry, the construction of a surface curve between n and sn (later on divided into volumetric upper and lower nasal height) helps to determine the morphological median sagittal line on which prn is located. Prn is defined as the point on the surface curve furthest from the direct line connecting n and sn (nasal height). It can be determined by applying a perpendicular length (effective nasal length) between the nasal height and the surface curve.
From here on, upper and lower volumetric nasal height can be used to align the mesh in the morphological median sagittal plane.	
Alar curvature base (acbR, acbL)	Most lateral point on the curved baseline of each alar. 3D anthropometrically, the points can be determined in anterolateral view (45° to each side of the front view) from the light reflection when the mesh is rotated around an imaginary longitudinal axis.
Alare (alR, alL; 2:46)	Most lateral point on each alar contour. 3D anthropometrically, the landmarks are set in the frontal view after alignment in the morphological median sagittal. Identical to acbR/acbL in most cases in infants, not in adults.
High points of columella (cR, cL, 3:25)	The point on each columella crest level with the top of the corresponding nostril. 3D anthropometrically, the landmarks are set in the anterocaudal view selecting a perpendicular view onto the columella.
Columellar median high point (cM 4:02)	Highest morphological median sagittal point on the columellar crest. 3D anthropometrically, the point can be identified at the intersection of the surface curves between cR and cL and between pr and sn.
Inner alare (aliR, aliL, 4:56)	Inner marking level at the most concentric midportion of the alae where the thickness of each ala is measured. The landmarks are situated at half-distance between the two attachment points of the alae, the anterior attachment point on the nasal body and the dorsal attachment point on the upper lip, respectively. 3D anthropometrically, the mesh should be positioned in caudal view to ensure correct positioning of the landmarks.
Outer alare (aloR, aloL, 4:56)	Outer marking level at the most eccentric midportion of the alae where the thickness of each ala is measured. The landmarks are situated at half-distance between the two attachment points of the alae, the anterior attachment point on the nasal body and the dorsal attachment point on the upper lip, respectively. 3D anthropometrically, the mesh should be positioned in caudal view to ensure correct positioning of the landmarks.
Subalare (sbalR, sbalL, 5:28)	Point at the lower limit of each alar base where it joins the skin of the upper lip and touches an imaginary tangent at the caudal base of the nose. 3D anthropometrically, the landmarks can be identified by the light reflection that moves along the alarm base when the mesh is rotated around a longitudinal axis.
Medial alar base (mabR, mabL, 6:03)	Point at the medial limit of each alar base where it meets the upper lip. 3D anthropometrically, the landmark is identified by following the light reflection that represents where the nose meets the facial/labial skin from the lateral to the most medial point. MabR/mabL is identical to cbR/cbL in alar base type 3.
Columella base (cbR, cbL, 6:40)	Most caudal and lateral point of the columella on each side at the bottom line where it meets the upper lip skin. 3D anthropometrically, the landmark is identified by following the light reflection that represents where the columella meets the labial skin from the medial to the most lateral point.
Cheilion (chR, chL, 7:10)	Point at each labial commissure. 3D anthropometrically, it may be helpful to follow the reflection and the difference in colour on the vermillion line or the reflection at the junction of the upper and lower lip to its most lateral point on each side.
Christa philtri (cphR, cphL, 7:54)	Point on each elevated margin of the upper lip, at the junction of the vermillion line and the white roll, representing the cranial extremes of the Cupid’s bow. 3D anthropometrically, the landmark is identified by the change in surface reflection on the vermillion to a continuous line on the vermillion edge when the mesh is rotated around a transversal axis in a slightly laterally angled view.
Labrale superius (ls, 8:30)	Caudal midpoint of the upper lip, at the junction of the vermillion line and the white roll, representing the caudal extreme of the Cupid’s bow. 3D anthropometrically, the landmark is identified at the junction of the previously identified reflection lines on the left and right vermillion edges.
Stomion (sto, 9:15)	Connection point between the upper and lower lip on the midline. 3D anthropometrically, it is defined as an imaginary point in the continuation of a vertical nasiolabial surface curve netween sn and ls (volumetric philtrum length) to the point where the upper lip touches the lower lip when slightly closed. In case of an open mouth scan, the landmark is created virtually at the estimated point of contact of the lips.
Vermillion-mucosal junction (vmjR/vmjL/vmjM, 10:00)	Vermillion-mucosal junctions below the cranial and caudal extremes of the Cupid’s bow. 3D anthropometrically, the landmark can be identified on a connecting surface curve between cphR/cphL/ls and stos at the point where the colour changes and the surface reflection simultaneously changes from inhomogeneous to homogeneous. This is best achieved by repeatedly rotating the mesh around the transverse axis.
Prolabiale (prl, 11:21)	Most protruded point on the upper lip in the morphological median sagittal. 3D anthropometrically, the landmark can be identified on the surface curve connecting sn and ls (volumetric philtrum length), with the distance between n and sn (nasal height) as the vertical reference plane, and chR and chL overlapping in the lateral view. AcP allows quantitative measurement of dimple depth.
Superior labial sulcus (sls, 12:24)	Deepest point of the superior labial sulcus (dimple). 3D anthropometrically, the landmark is identified as the point furthest from a direct line connecting sn and prl on the surface curve between sn and prl/ls.
Lateral vermillion (lvR, lvL, 13:17)	Most protruded point of the vermillion convexity. 3D anthropometrically, the landmark can be identified as the point furthest from a connecting line between chR/chL and ls (right and left vermillion length) on the vermillion edge reflection previously identified when setting cphR, cphL and ls.

**Table 2:** Overview and definition of the 3D anthropometric measurements consisting of of 32 distances (Fig. 1b), 33 surface curves (Fig. 1c), 2 angles (Fig. 1d) and the resulting 18 indices.

3D Measurements	Definition
Distances (corresponding number in Fig. 1)	Direct length between two landmarks or between a landmark and a point on a previous measurement
Nasal height (D1)	n-sn
Nasal base width (D2)	acbR-acbL
Nasal alar width (D3)	alR-alL
Effective nasal length (D4)	Shortest distance (perpendicular length) between prn and a connecting line between n and sn (nasal height).
Right alar base to pronasale (D5)	acbR-prn
Left alar base to pronasale (D6)	acbL-prn
Right alar thickness (D7)	aloR-aliR
Left alar thickness (D8)	aloL-aliL
Caudal nasal base width (D9)	sbalR-sbalL
Right nostril floor width (D10)	mabR-cbR
Left nostril floor width (D11)	mabL-cbL
Right lateral columella length (D12)	cR-cbR
Left lateral columella length (D13)	cL-cbL
Median sagittal columella length (D14)	cM-sn
Mouth width (D15)	chR-chL
Dimple depth (D16)	Shortest distance (perpendicular length) between sls and a connecting line between acp and sn.
Vertical heights of the right lateral lip (D17)	sbalR-cphR
Vertical heights of the left lateral lip (D18)	sbalL-cphL
Vertical heights of the right medial lip (Philtrum) (D19)	cbR-cphR
Vertical heights of the left medial lip (Philtrum) (D20)	cbL-cphL
Upper lip height (D21)	sn-sto
Philtrum length (D22)	sn-ls
Right vermillion length (D23)	chR-ls
Left vermillion length (D24)	chL-ls
Transverse lengths of the right lateral lip element (D25)	chR-cphR
Transverse lengths of the left lateral lip element (D26)	chL-cphL
Right vermillion convexity (D27)	Shortest distance (perpendicular length) between lvR and a connecting line between chR and cphR (transverse lengths of the right lateral lip element).
Left vermillion convexity D28)	Shortest distance (perpendicular length) between lvL and a connecting line between chL and cphL (transverse lengths of the left lateral lip element).
Right lateral mouth to nose distance (D29)	chR-acbR
Left lateral mouth to nose distance (D30)	chL-acbL
Right median-sagittal mouth to nose distance (D31)	ls-cbR
Left median-sagittal mouth to nose distance (D32)	ls-cbL
**Surface curves** (corresponding number in Fig. 1)	**Curve between two landmarks positioned on the surface of the mesh (raw placement of surface curves without intersection point is indicated on the measurements with no further comments)**
Volumetric upper nasal height (S1)	n-prn
Volumetric lower nasal height (S2)	prn-sn
Volumetric nasal height (S3)	n-prn-sn
Volumetric soft-tissue nasal width right (S4)	acbR-prn
Volumetric soft-tissue nasal width left (S5)	prn-acbL
Right inner nostril circumference (S6)	Inner nostril circumference starting and ending at cbR; measured on the most concentric portion of the nostrils touching cbR, mabR and aliR.
Left inner nostril circumference (S7)	Inner nostril circumference starting and ending at cbL; measured on the most concentric portion of the nostrils touching cbL, mabL and aliL.
Right volumetric lateral columella length (S8)	Connecting cR-cbR on the columella crest; part of the right inner nostril circumference.
Left volumetric lateral columella length (S9)	Connecting cL-cbL on the columella crest; part of the left inner nostril circumference.
Right columellar base width (S10)	cbR-sn
Left columellar base width (S11)	sn-cbL
Columellar base width (S12)	cbR-sn-cbL
Volumetric philtrum length (S13)	sn-ls
Philtrum base width (Volumetric) (S14)	Connecting cphR-ls-cphL on the cranial vermillion borders light reflection.
Medial vermillion height (S15)	ls-vmjM-stos
Right vermillion height (S16)	cphR-vmjR-stos
Left vermillion height (S17)	cphL-vmjL-stos
Volumetric upper lip height (S18)	sn-ls-stos
Right dry vermillion height (S19)	cphR- vmjR
Median dry vermillion height (S20)	ls- vmjM
Left dry vermillion height (S21)	cphL- vmjL
Right wet vermillion height (S22)	vmjR-sto
Median wet vermillion height (S23)	vmjM-sto
Left wet vermillion height (S24)	vmjL-sto
Right volumetric vermillion length (S25)	chR-ls measured on the cranial vermillion borders light reflection touching cphR.
Left volumetric vermillion length (S26)	chL-ls measured on the cranial vermillion borders light reflection touching cphL.
Vertical heights of the right medial lip (Philtrum, Volumetric) (S27)	Connecting cbR-cphR along the philtrum edge
Vertical heights of the left medial lip (Philtrum, Volumetric) (S28)	Connecting cbL-cphL along the philtrum edge
Right philtrum base width (Volumetric) (S29)	Connecting cphR-ls on the cranial vermillion border. 3D anthropometrically, the surface curve is placed at the switch in light reflection when the mesh is rotated around an imaginary longitudinal axis.
Left philtrum base width (Volumetric) (S30)	Connecting ls-cphL on the cranial vermillion border. 3D anthropometrically, the surface curve is placed at the switch in light reflection when the mesh is rotated around an imaginary longitudinal axis.
Right lateral volumetric vermillion length (S31)	chR-cphR measured on the cranial vermillion borders light reflection.
Left lateral volumetric vermillion length (S32)	chL-cphL measured on the cranial vermillion borders light reflection.
**Angles** (corresponding number in Fig. 1)	**3D anthropometric angles as three-point angles or two-directional angles**
Columellar angle (A1)	Angle between cM-sn as first direction and the horizontal plane created by connection of the commissures chR-chL as second direction (two-directional angle)
Vermillion angle (A2)	chR-ls-chL with ls as angle point (three-point angle)
**Indices**	
Vertical nasal convexity index	n-prn-sn / n-sn (surface curve div. by direct distance) volumetric nasal height/nasal height
Alar convexity index	(acbR-prn + acbL-prn) / acbR-prn-acbL (surface curves div. by direct distance) (volumetric soft-tissue nasal width right + volumetric soft-tissue nasal width left) / (right alar base to pronasale + left alar base to pronasale)
Right columella index	cR-cbR / cR-cbR (surface curve div. by direct distance) right volumetric lateral columella length / right lateral columella length
Left columella index	cL-cbL / cL-cbL (surface curve div. by direct distance) left volumetric lateral columella length / left lateral columella length
Columella index	(cR-cbR + cL-cbL) / (cR-cbR + cL-cbL) (surface curves div. by direct distance) (right volumetric lateral columella length + left volumetric lateral columella length) / (right lateral columella length + left lateral columella length)
Horizontal nasal convexity index	(acbR-prn + prn-acbL) / acbR-acbL (surface curves div. by direct distance) (volumetric soft-tissue nasal width right + volumetric soft-tissue nasal width left) / nasal base width
Nasal convexity index	VNCI + HNCI / 2 (vertical nasal convexity index + Horizontal nasal convexity index) / 2
Columella base symetry index	cbR-sn / sn-cbL (surface curves div. by surface curve) right columellar base width / left columellar base width
Dimple index	sn-ls / sn-ls (surface curve div. by direct distance) volumetric philtrum length / philtrum length
Philtrum taper index	cphR-ls-cphL / cbR-sn-cbL (surface curves div. by surface curve) philtrum base width (Volumetric) / (right columellar base width + left columellar base width)
Upper lip index	sn-ls-stos / sn-stos (surface curve div. by direct distance) volumetric upper lip height / upper lip height
Right vermillion index	chR-ls / chR-ls (surface curve div. by direct distance) right volumetric vermillion length / right vermillion length
Left vermillion index	chL-ls / chl-ls (surface curve div. by direct distance) left volumetric vermillion length / left vermillion length
Vermillion index	(chR-ls + chL-ls) / (chR-ls + chL-ls) (surface curves div. by direct distance) (right volumetric vermillion length + left volumetric vermillion length) / (right vermillion length + left vermillion length)
Lateral mouth to nose symetry index	(chR-acbR + cphR-sbalR) / (chL-acbL + cphL-sbalL) (direct distances div. by direct distances) (right lateral mouth to nose distance + vertical heights of the right lateral lip) / (left lateral mouth to nose distance + vertical heights of the left lateral lip)
Central mouth to nose symetry index	(cphR-cbR + ls-cbR) / (cphL-cbL + ls-cbL) (direct distances div. by direct distances) (vertical heights of the right medial lip (Philtrum) + right median-sagittal mouth to nose distance) / (vertical heights of the left medial lip (Philtrum) + left median-sagittal mouth to nose distance)
Mouth to nose symetry index	(LMTNSI + CMTNSI) / 2 lateral mouth to nose symetry index + central mouth to nose symetry index / 2
Soft-tissue mouth to nose index	chR-chL / (alR or acbR-alL or acbL depending on which is the most lateral point) (direct distances div. by direct distances)

A distance is defined as the direct length between two points on a mesh or between a point on the mesh and a point on a previous measurement. A distance between a previous measurement and a new landmark can be constructed as a perpendicular length, as in the example of effective nasal length, dimple depth or right and left vermillion convexity (Fig. 1b).

Unlike normal curves, surface curves are positioned directly on the mesh (Fig. 1c). They are used to generate curvature curves that reflect the subject’s natural anatomy, while remaining independent of mesh/face rotation in space. This was confirmed by the authors through repetitive measurements using surface curves while manipulating the respective meshes in space, as well as by consulting the software developers. As a fundamental advance in the transition from conventional to 3D anthropometry, surface curves provide the ability to make new and more comprehensive statements about the facial morphology. They include the previously overlooked anatomical configurations between their respective start and end points. Taking into account the specific characteristics of each parameter and its prospective integration in future studies of cleft patients, we have determined for each surface curve, in particular, whether the intersection points are valid. If no intersection points are valid, only the start and end points of the surface curve should be set manually. Detailed instructions for each parameter are given in the Supplementary Material section.

3D anthropometric angles can be constructed in two ways: as three-point angles or as bipartite angles (Fig. 1d). Three-point angles are defined by three landmarks, one of which acts as the angle point. Bipartite angles can be constructed using elements containing a direction, a normal or a line (e.g. planes and lines) without them having to touch. In the context of our 3D anthropometric methodology, the vermillion angle is constructed as a 3-point angle between right and left cheilion and labrale superius, with the latter acting as the angle point. As a 2-directional angle, the columellar angle is formed by the line/distance between columellar median high point and subnasale, and the line/distance between right and left cheilion.

The indices function as constructed variables, facilitating the formation of new assertions regarding the interrelationship between the parameters and allowing a rapid assessment of the patient’s nasolabial conditions.

The data set was analysed according to 3D anthropometric criteria by metrically accurate measurements of distances, surface curves, angles and indices using 3D inspection software (GOM Inspect 2019 Hotfix 6, Rev. 125216, Build 2020-02-27, Co. Zeiss, Jena, Germany).

### Statistical analysis

Reliability tests were conducted using a sub-collective as a proxy for the entire collective to ensure reliable measurements. With an interval of two weeks intra-rater reliability was assessed for five children. Intra-rater reliability was calculated using the time points as different assessment occasions. The two-way intraclass correlation coefficient (ICC) for consistency and Pearson’s correlation coefficient (r) were calculated as measures of intra-rater reliability. Inter-rater reliability is evaluated across all children for five parameters between three raters using the intraclass correlation coefficient (ICC) for two-way models employing an absolute agreement definition^21,22^. To ensure a high degree of reliability in the test, the working group mixed presumably ’easy’ and ‘hard’ parameters, as well as parameters relating to the nose and lip. Distances and surface curves of each kind were included. An ICC of < 0.40 is as considered poor agreement, values between 0.40 and 0.75 as substantial- and an ICC exceeding 0.75 as excellent^23^.

To improve the accuracy of the data, values were imputed based on clinically relevant information, such as the subject’s sex, age, and birth weight. This led to the predicted values presented in the Results section. Rationale for the imputation process: Providing the algorithm with high quality data from a limited number of samples, which have been measured with precision by an expert in the field, is a time-consuming process, but is preferable to measurements made by an expert under time pressure or by a non-expert for a large number of samples. In the present study, valid and precise data are employed to predict nonobserved values, rather than providing additional clinical data within the same time frame. This approach aims to minimize the impact of human error. All variables for which quantitative data were available were used to predict explicit missing values using the R package Multivariate Imputation by Chained Equations (mice 3.15.0) with predictive mean matching. Predictive mean matching was chosen because it has been shown to be effective in dealing with nonlinearity and outliers. An example of the observed and predicted parameters for 9 direct lengths and 7 surface curves is given in Table 3.:

**Table 3:** Example for observed and predicted parameters for 9 direct lengths and 7 surface curves. All values are given in mm.

Parameter		M/Md ± SD	
		Observed (*n* = 25)	Predicted (*n* = 202)
Length (l)/surface curve (s) in mm	1. Right vermillion length (l)	19.8/19.7 ± 1.99	19.6/19.7 ± 2.11
	2. Left vermillion length (l)	19.8/20.1.1 ± 2.22	19.3/19.3 ± 1.73
	3. Right lateral volumetric vermillion length (s)	18.4/18.5 ± 1.93	18.8/18.4 ± 2.34
	4. Left lateral volumetric vermillion length (s)	18.1./17.7 ± 2.25	17.9/17.8 ± 1.69
	5. Right lateral columella length (l)	5.5/5.3 ± 1.13	5.4/5.3 ± 1.17
	6. Left lateral columella length (l)	5.5/5.6 ± 1.11	5.3/4.9 ± 1.16
	7. Effective Nasal length (l)	8.3/8.4 ± 0.90	8.0/8.4 ± 1.13
	8. Right volumetric lateral columella length (s)	6.9/6.4 ± 1.85	7.2/7.1 ± 1.59
	9. Left volumetric lateral columella length (s)	6.7/6.6 ± 1.52	6.7/6.4 ± 1.72
	10. Median sagittal columella length (l)	5.6/5.6 ± 1.03	5.8/5.7 ± 0.80
	11. Philtrum length (l)	11.4/11.1 ± 1.26	11.1/10.8 ± 0.84
	12. Right volumetric vermillion length (s)	22.5/22.9 ± 2.16	22.9/23.3 ± 2.33
	13. Left volumetric vermillion length (s)	22.4/22.5 ± 2.50	22.8/22.5 ± 2.68
	14. Transverse lengths of the right lateral lip element (l)	17.6/17.8 ± 1.82	17.9/18.0 ± 1.44
	15. Transverse lengths of the left lateral lip element (l)	17.3/17.1 ± 2.06	17.3/17.1 ± 1.68
	16. Volumetric philtrum length (s)	11.7/11.4 ± 1.37	11.4/11.1 ± 1.04

The table shows the differences between observed and predicted values for selected distances and surface curves to illustrate the rationale behind the imputation.

The dataset used to impute missing values consisted of 25 children, with each subject having 85 distinct variables. The age range used for imputation was 3 to 8 months and 30 days, respectively. For the purposes of this analysis, each month was assumed to consist of 30 days. For the age range of 5 months (5/0–5/30 months/days) only one measurement point was available (5/22 months/days). The child was included in the dataset, but the age range of five months was not predicted. Similarly, for the age of seven months, there was no data available for a child and therefore the range was not predicted.

Parameters that are likely to be highly correlated, such as length measurements, were approximated together and grouped by individual. The rationale is once more straightforward:

It can be assumed that the “left lateral columella length” corresponds to the “right lateral columella length” of a healthy individual. Yet, for the purposes of this approximation, it is not necessary for this axiom to be met. However, if the aforementioned axiom is proven to be true, the information is utilised. It is important to note that the data are inherently interdependent, as some indices are calculated from the raw values of other variables. In addition, the values are naturally dependent on age, sex and birth weight. Consequently, if a raw value is missing, the index cannot be calculated, resulting in a dependent pattern of missing data. It should be noted that subsequently Little’s MCR Test was not computed.

Statistical analysis led to the presentation of the results in the form of growth curves and formulae according to Brons et al., Matthews et al. and Liang et al.^24–26^.

## Results

Our final reference database consists of 32 distances (Fig. 1b), 32 surface curves (Fig. 1c), two angles (Fig. 1d) and 18 indices; representing the optimal morphological outcome as a control group for future research in cleft lip and palate surgery. Descriptive plots of all 84 datasets can be found in Supplementary Material 2, with observed values on the left and predicted values on the right. Age is given in months on the x-axis, and the specific value for each parameter is given on the y-axis in mm for distances and surface curves, in degrees for angles, and in numbers without dimension for indices. The red lines represent growth curves for female individuals and the blue lines for male individuals. To facilitate the application in both clinical and research settings, the comprehensive mathematical equation for each parameter is provided in Supplementary Material 3, with the equations presented for both male and female patients. In order to determine the optimal morphological result for a particular parameter, it is necessary to enter the current clinical value as age in months in the equation, or for simplification purposes in the column provided for the respective sex. We recommend using the predicted value equations.

For each child and each parameter, the intra-rater reliability was good/high or excellent/very high with a minimum value of ICC = 0.89 and *r* = .89. Stratified by type of parameter intra-rater reliability is lowest for angles and highest for surfaces (ICC = 0.99) with distances ranging in between (ICC = 0.90). For a tolerance of 1 mm, percentage agreement across all indices and children was 81.4%. Across variables assessed ICC for inter-rater reliability was 0.99 (*p* < .001) indicating nearly perfect absolute agreement between raters. The lowest ICC was found for the right vermillion length with an ICC of 0.73 (*p* < .001) indicating substantial agreement among raters. For a tolerance of 1 mm percentage agreement between raters was 43.9%. A possible cause of the lower ICC for some parameters, including right vermillion length, could be the challenging placement of the cheilion points.

Accurate positioning of the updated 3D anthropometric landmarks is required to ensure reproducible measurements. Therefore, in addition to a step-by-step tabular guide (Table 1 and Supplementary Material 1), we present a short video guide for 3D anthropometric landmark positioning (Supplementary Material 4).

As part of the establishment of our 3D anthropometric measurements and conclusive reference database, the new methodology allowed for a dedicated classification of alar and nasal shape. To facilitate future studies and to improve the individualised surgical approach to cleft lip and other nasolabial surgical procedures in general, we have identified the following three types of alar base:

- Type I: mabR/mabL is more lateral than cR/cL.
- Type II: mabR/mabL is more medial than cR/cL.
- Type III: Direct transition from mabR/mabL to cbR/cbL.

**Fig. 2:**
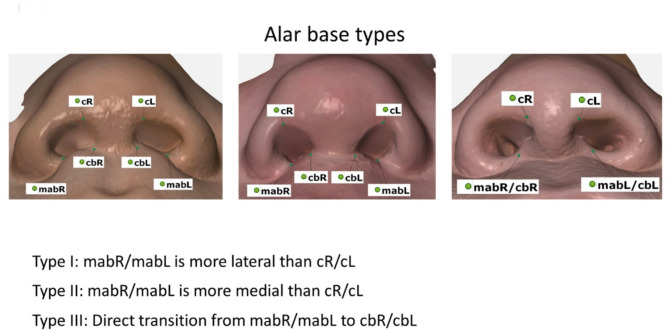
Alar base types. Type I: mabR/mabL is more lateral than cR/cL. Type II: mabR/mabL is more medial than cR/cL. Type III: Direct transition from mabR/mabL to cbR/cbL.

Alar base type 1, the most common in our study population, is characterised by a wide and shallow labial nasal entrance and a continuously narrow columella. Alar base type 2 is characterised by a narrow and shallow labial nasal entrance and a columella that extends far cranially/anteriorly. In alar base type 3, a continuous ridge shields the labial nasal entrance from the rest of the upper lip. As shown in Fig. 2, alar base types not only influence the appearance of the alar, but also have a major impact on the overall appearance of the patient’s nose and can therefore be used to classify the nose in general.

In addition to the previous validation of metric accuracy of the intraoral scanner^12^we can now confirm its clinical applicability based on the long-term use in awake and sleeping children aged 3 to 9 months.

## Discussion

To facilitate future studies in cleft surgery, we have identified the optimal morphological endpoint in the nasolabial region of healthy children aged 3 to 9 months. Our reference database will allow clinicians and research groups to compare the status quo or the morphological baseline of cleft patients before and after cleft lip repair with a healthy control group. Similar to the description of bone growth in healthy children by Liang et al., we present growth curves that now allow graphical and mathematical prediction of the expected value at a given age and sex^25^. In addition to previously established anthropometric parameters of the nasolabial region^20^our database takes into account recent advances in 3D anthropometry and includes surface curves, perpendicular lengths and the resulting comprehensive indices. To our knowledge, this is the first 3D anthropometric database of the nasolabial region in healthy children aged 3 to 9 months.

As mentioned above, 3D anthropometry is a highly accurate and advanced tool for evaluating the nasolabial region and has been shown to be superior to two-dimensional methods^27,28^. In line with this statement, recent literature indicates a paradigm shift in the evaluation of CLP surgery towards 3D evaluation^13,15,18,29–32^. To date, the majority of recent studies lack validation of the 3D (facial) scanner used^13,15,18,30,31^ and it is worth noting that some of the scanners used do not achieve the accuracy for craniofacial assessment touted by the industry^12^. As a 3D scanner for the assessment of cleft lip and palate must not only be highly accurate but also mobile in order to be used in clinical applications, a suitable device has recently been validated by our working group using a previously validated industrial scanner as a baseline^12^.

The use of this new data acquisition tool has opened up a host of new possibilities for 3D data processing and measurement. The mere ability to rotate the meshes in 3D space, together with a constant virtual light source, enables the accurate identification of previously difficult or indistinguishable anatomical structures through light reflection and shading. As light reflection and shading occur naturally at the edges of 3D structures in space and have been shown to be suitable for the perception and detection of 3D structures^33–36^the reflections appear to be well suited for the detection and separation of anatomical structures in the nasolabial region, such as the nose from the facial and labial tissues, the alae from the alar base, and the vermillion from the upper lip (see video and tables in the supplementary material). Furthermore, both our own experience and the current literature indicate that 3D anthropometry on coloured 3D surfaces seems to benefit 3D shape perception^36^. As most of the current articles on 3D anthropometry of the nasolabial region include measurements on monochrome surfaces^32,37–39^we recommend their implementation for further research in our field. It is worth mentioning, as a limiting factor, that although general data reproducibility was high to very high, some landmarks were slightly less reproducible than others with regard to inter-rater reliability. For instance, this was the case for parameters including the left and right cheilion as landmarks. These limitations could be overcome through future research and technological advances.

The novel approach of combining precise measuring instruments with the use of light reflections on coloured 3D surfaces now paves the way for a new and accurate method of measuring the outcome of cleft lip plasty.

Self-critical examination of one’s own accuracy and, above all, the reproducibility of data collection and processing is an important part of science and one that has been increasingly emphasised in recent years^40,41^. As we are now on the threshold of a paradigm shift towards 3D evaluation of cleft lip surgery with the implementation of all its technical and methodological possibilities, a unified and standardised approach within the discipline and between the working groups is of great importance. To this end, in addition to the reference database, we present step-by-step instructions for the consistent and reproducible placement of the updated anthropometric landmarks of the nasolabial region in text and video. Once the 33 landmarks have been created, the children (i.e. their scans) are measured using distances, surface curves, angles and indices. Once again, the use of 3D anthropometric methods offers new and far-reaching possibilities in all four areas of application mentioned, which go beyond their pure superiority in terms of precision and applicability.

The concept of distance has expanded beyond its traditional definition. It now encompasses more than just the linear measurement between two points (A and B). In modern spatial analysis, distance can also be quantified as the perpendicular length from a reference line to a point on a mesh or virtual surface. This technique, which to our knowledge has never been used in 3D nasolabial anthropometry, was used by our working group to determine the parameters of effective nasal length, dimple depth and right and left vermillion convexity.

When measuring the distance between point (A) and point (B), we disregard all information about the morphology in the form of natural convexities and concavities of the nasolabial region between the two points. Therefore, with the new capabilities of 3D anthropometry, we introduce the entity of surface curves, which follow the patient’s natural anatomy on the surface of the mesh, allowing it to be included in future evaluations and research.

Vermillionplasty is essential for adequate closure of the lip in unilateral cleft lip. Various techniques, with Noordhoff’s technique as a prominent forerunner, are currently being discussed with regard to their effect on vermillion convexity and, in the case of a suboptimal result, the loss of vermillion convexity^1,42,43^. Similarly, different techniques for bilateral cleft lip closure and their respective effects on the dimple morphology, which strongly influences the perception of a person’s attractiveness, have been the subject of much debate^1,44–47^. By quantifying the aforementioned distances, in particular dimple depth and left and right vermillion convexity, and adding surface curves, in this case the volumetric philtrum and vermillion length, as a new entity, we allow for profound and far-reaching conclusions to be drawn in future studies on the impact of different techniques in cleft lip surgery.

The pursuit of reproducible data collection and processing should go beyond the implementation of accurate and precise measuring methods. It is also essential to use a reliable statistical approach. Inclusion of missing data is used in several research areas where resources such as expertise, cost or readily available samples are scarce^48,49^. As long as the prediction model does not become too complex (at present), it has been shown that clinical data can be imputed reliably. For example, using data from 13 common laboratory tests and deliberately removing known information, 12 international teams were able to correctly estimate the missing information using probabilistic algorithms^50^. Clinical research needs to revalue missing values not as a genuine problem of a dataset, but as a challenge for mathematical modelling given the probabilistic and Bayesian algorithms available. Simply treating missing information as not present can lead to more severe artefacts in statistical analysis (e.g. assuming non-normality where data are actually normally distributed, up-weighting of actually random occurrences, which enlarges the confidence interval) than treating them^51^.

**Fig. 3:**
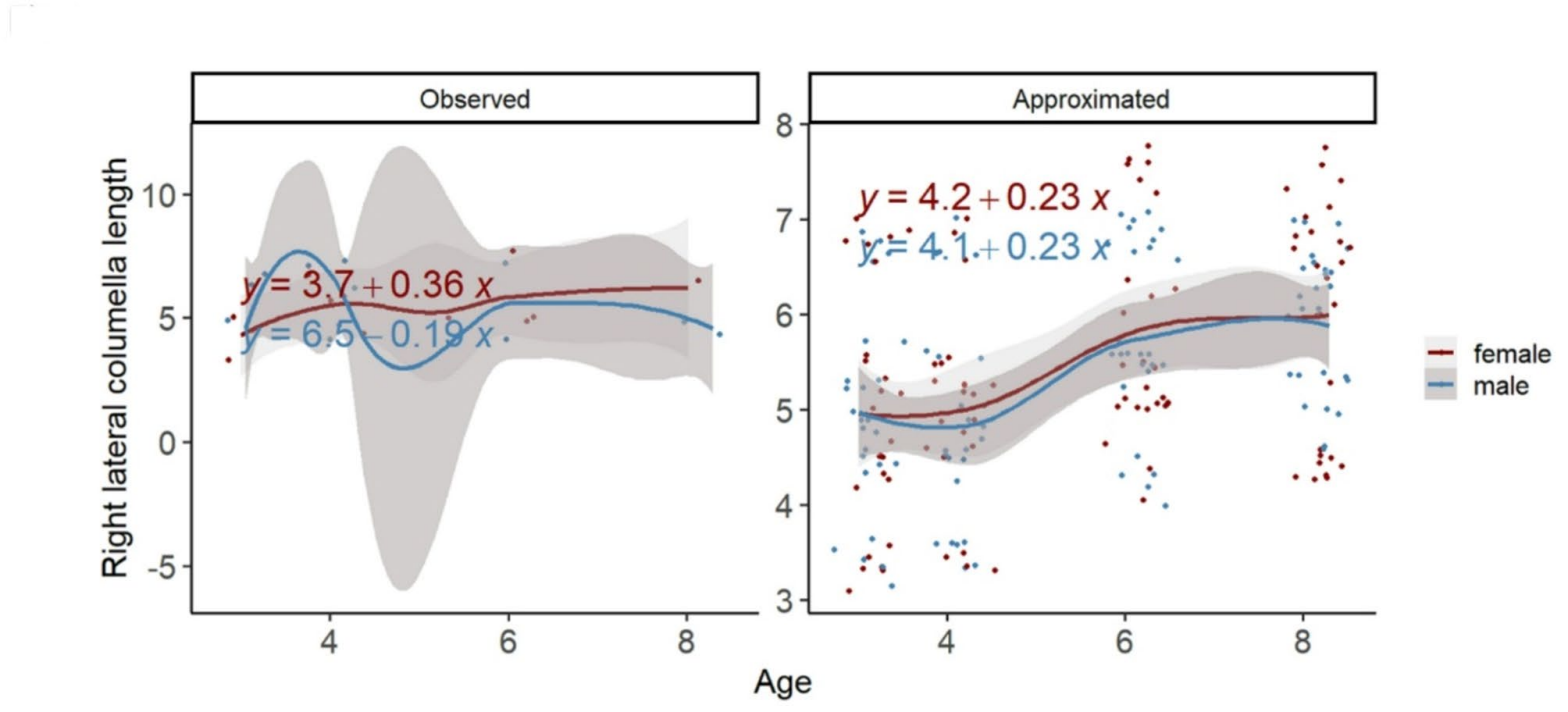
Observed and predicted values based on age, sex and weight for right lateral columnella length. All values are given in months and mm. The observed information leads the equation to assume a decrease in males, based on the large confidence interval between four and six months. However, the multiple chained regression equations for the predicted values indicates growth, thereby reducing the size of the confidence interval and providing information on a more fine-grained y-axis. Y-axis shows distance (mm). X-axis shows age (months).

In our case, the benefit of imputation can be demonstrated by examining, for example the length of the right lateral columella (Fig. 3). The observed information leads the equation to assume a decrease in males, based on the large confidence interval between four and six months. However, the multiple chained regression equations for the predicted values indicates growth, thereby reducing the size of the confidence interval and providing information on a more fine-grained y-axis.

The morphology of the nose, in particular the nostrils and their attachment point to the upper lip and the aforementioned columella, has a major influence on the overall appearance of an individual. Leslie G. Farkas has previously classified alar base configurations based on visually assessed criteria such as ‘short curved’ and ‘full curved’^20^. Despite the new 3D anthropometric capabilities, modern classifications are limited to pathologies and different treatment methods without describing the optimal morphological endpoint^52–55^. We therefore propose a simple classification of alar base types based on the 3D anthropometric landmarks cR/cL, cbR/cbL and mabR/mabL and their spatial relationship (Fig. 2). The potential for a detailed and consistent characterisation of alar base types now allows them to be incorporated into surgical procedures for the treatment of cleft lip. This can be achieved by considering the healthy side of the nose in unilateral clefts, or by considering the alar base types of close relatives. It should be noted, however, that further work on the inheritance of alar base types is needed to make accurate predictions based on close relatives.

Despite the high drop-out rate as a limiting factor of the study, it seems important to lay an early foundation in the form of reference databases on the nasolabial region of healthy children aged 3–9 months, thus anticipating technical advances in clinical 3D anthropometry. By minimising the time required per scan, intraoral scanners may become more feasible for awake and active children in the future. The use of smartphone-based scans also offers promising approaches, despite losses in metric accuracy^56–58^.

Good coverage rates and confidence interval widths can be achieved with small samples when suitable donor cases are available for imputation. The suitability of donor cases is inevitably linked to the (clinical) population of interest, addressing the underrepresentation of different ethnic groups. Although children of Asian (*n* = 2), Arab (*n* = 4) and African (*n* = 1) ethnicity were included to represent the average Central European patient population, there is no specific ethnic analysis of the optimal morphological endpoint. Given the small sample size and high exclusion rate despite precise measurement methodology, this should be the subject of future studies. The generation of ethnicity-specific databases, and the resulting potential to improve the treatment of nasolabial defects for individual patients, represents a challenging yet promising area of research.

## Conclusions

By defining the optimal morphological endpoint, we have successfully established a basis for future 3D anthropometric measurements in children with cleft lip and palate. The establishment of a reference database and a step-by-step tutorial will allow reproducibility of measurements in future studies and facilitate the comparison of the clinical status quo with the optimal morphological endpoint. In addition, alar base types based on 3D anthropometric measurements may allow for a more individualised surgical approach to cleft lip and palate treatment.

## Electronic supplementary material

Below is the link to the electronic supplementary material.

Supplementary Material 1

Supplementary Material 2

Supplementary Material 3

Supplementary Material 4

Supplementary Material 5

## Data Availability

All data generated or analysed during this study are included in this published article (and its Supplementary information files).

## Abbreviations

3D: 3-dimensional
FS: facial scanner
IOS: intraoral scanner
CLP: cleft lip and palate
STL: stereolithography
