# Supplementary material for: 3D anthropometry of the nasolabial region in children aged 3 to 9 months as reference database for clinical assessment
Source: Sci Rep. 2025 Jul 28;15:27443. doi: 10.1038/s41598-025-11024-8 (PMC12304160; doi:10.1038/s41598-025-11024-8)
Supplement: Supplementary file 1 — Supplementary Material 1 [file 41598_2025_11024_MOESM1_ESM.docx]

| **Landmarks**  (abbreviation,  timestamp for  video tutorial, corresponding number in Figure 1) | **Definition** |
| --- | --- |
| Nasion (n; 0:02) | Point in the midline of the nasal root identical to the hard tissue nasion. 3D anthropometrically, the n point can be identified at the point of change in light reflection by repeatedly rotating the mesh around an imaginary transverse axis. 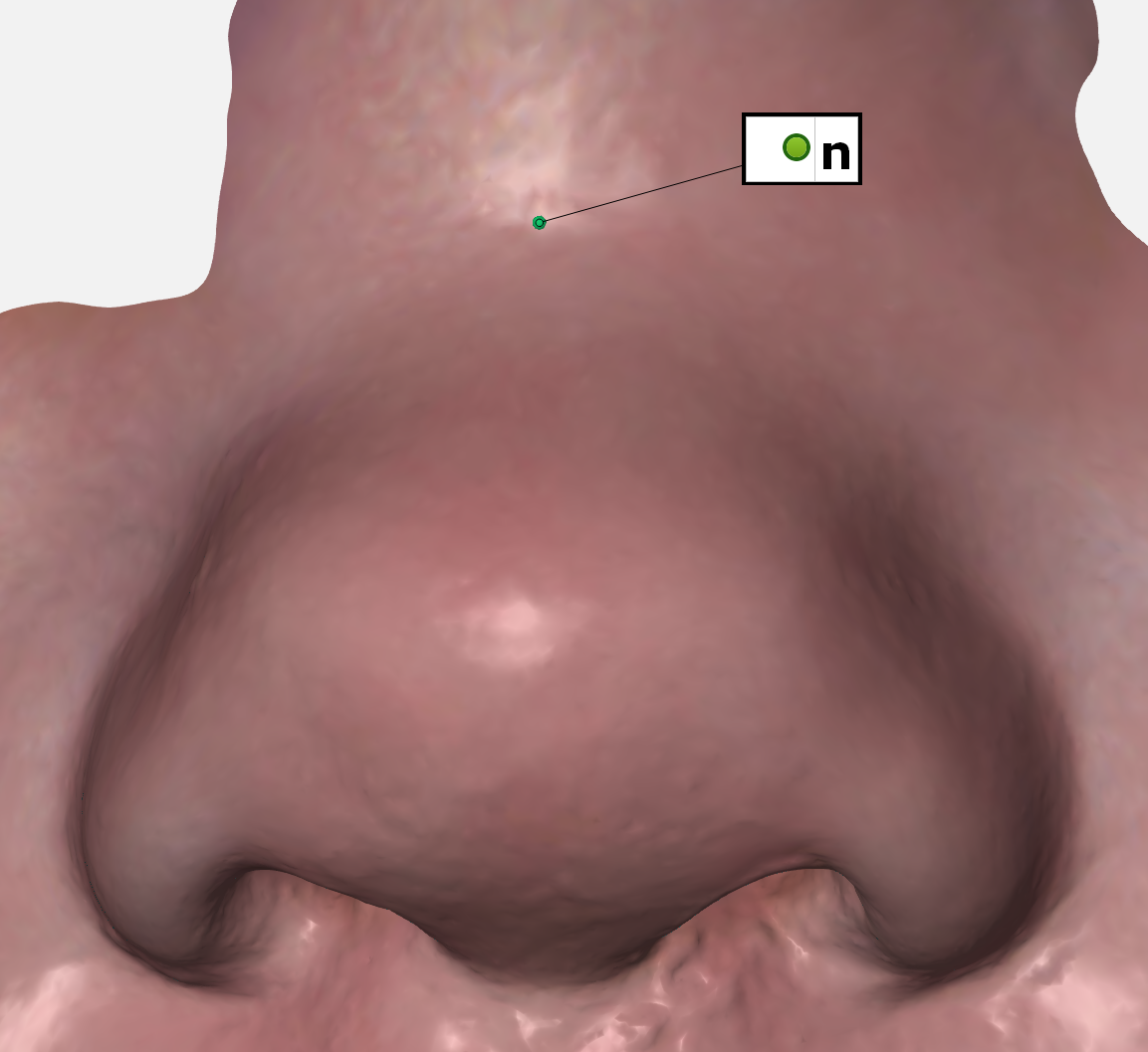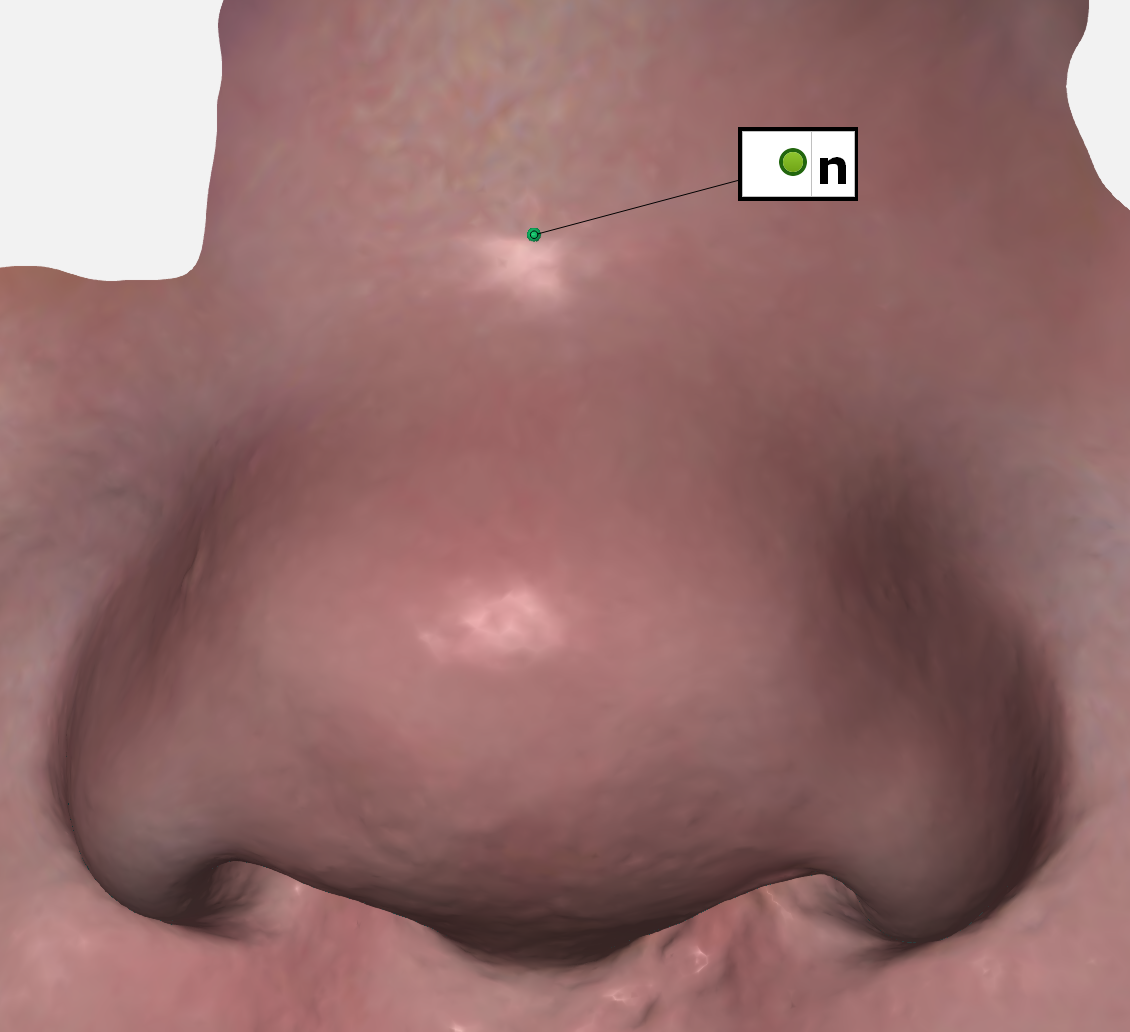 |
| Subnasale (sn; 0:40) | Midpoint of maximum concavity where the upper lip skin meets the columella base. 3D anthropometrically, the landmark can be located based on the light reflection at the caudal columellar base on an imaginary line connecting prn and ls.  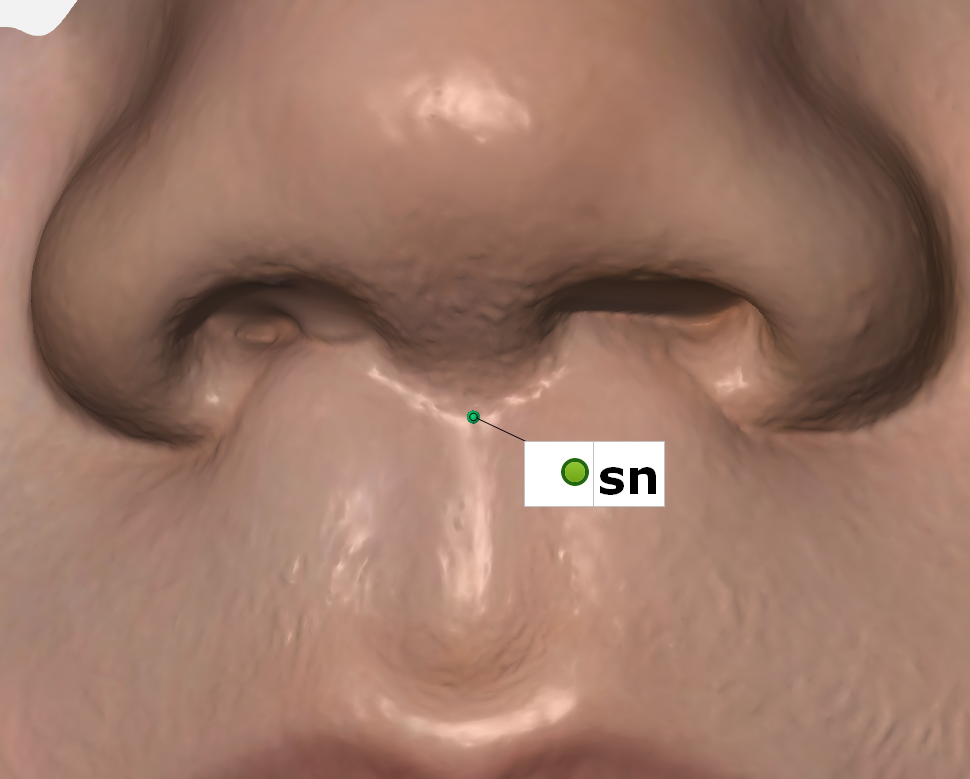 |
| Pronasale (prn; 1:23) | The most prominent point of the nasal tip on the morphological median sagittal line. In 3D anthropometry, the construction of a surface curve between n and sn (later on divided into volumetric upper and lower nasal height) helps to determine the morphological median sagittal line on which prn is located. Prn is defined as the point on the surface curve furthest from the direct line connecting n and sn (nasal height). It can be determined by applying a perpendicular length (effective nasal length) between the nasal height and the surface curve.  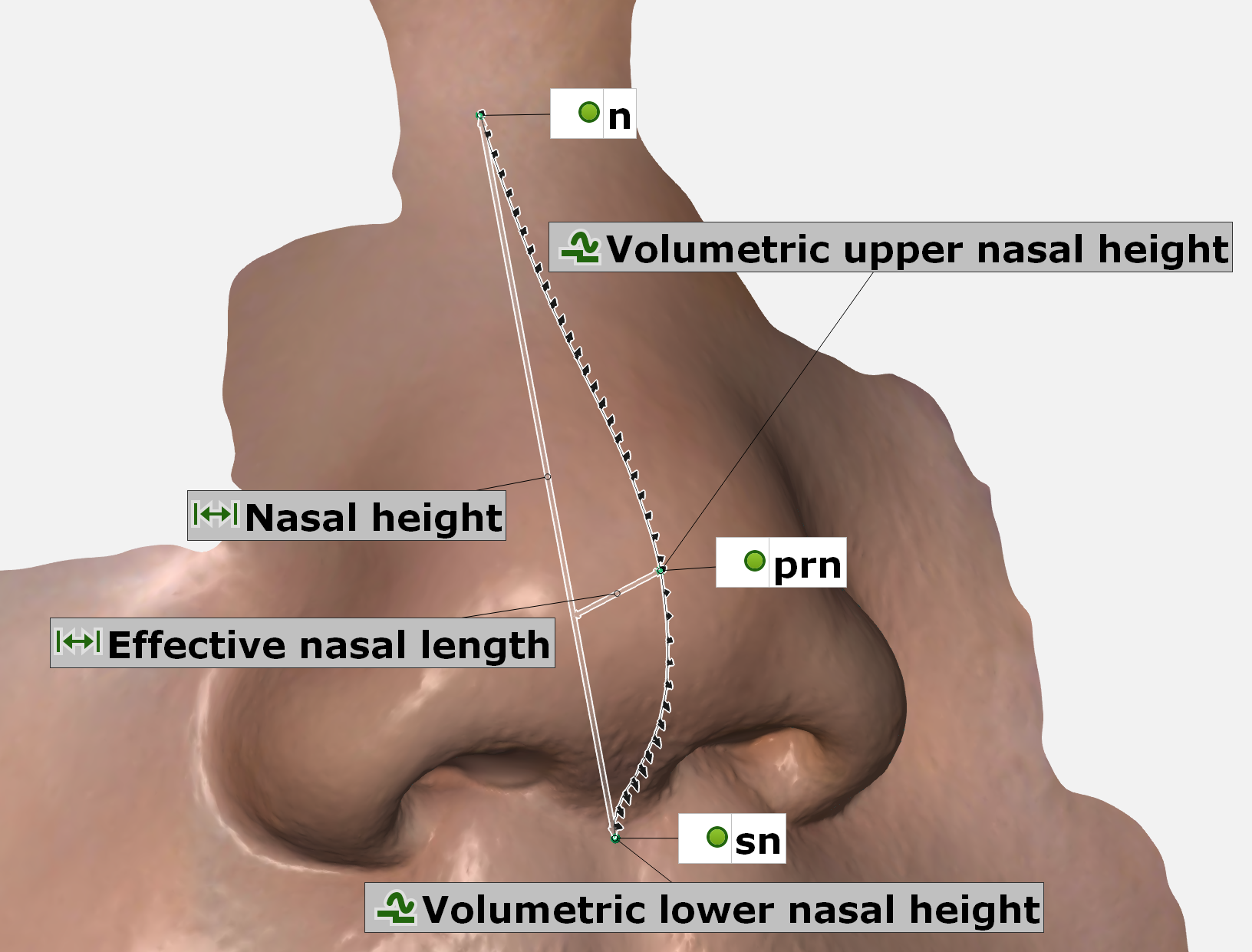 |
| From here on, upper and lower volumetric nasal height can be used to align the mesh in the morphological median sagittal plane. | |
| Alar curvature base (acbR, acbL) | Most lateral point on the curved baseline of each alar. 3D anthropometrically, the points can be determined in anterolateral view (45° to each side of the front view) from the light reflection when the mesh is rotated around an imaginary longitudinal axis.  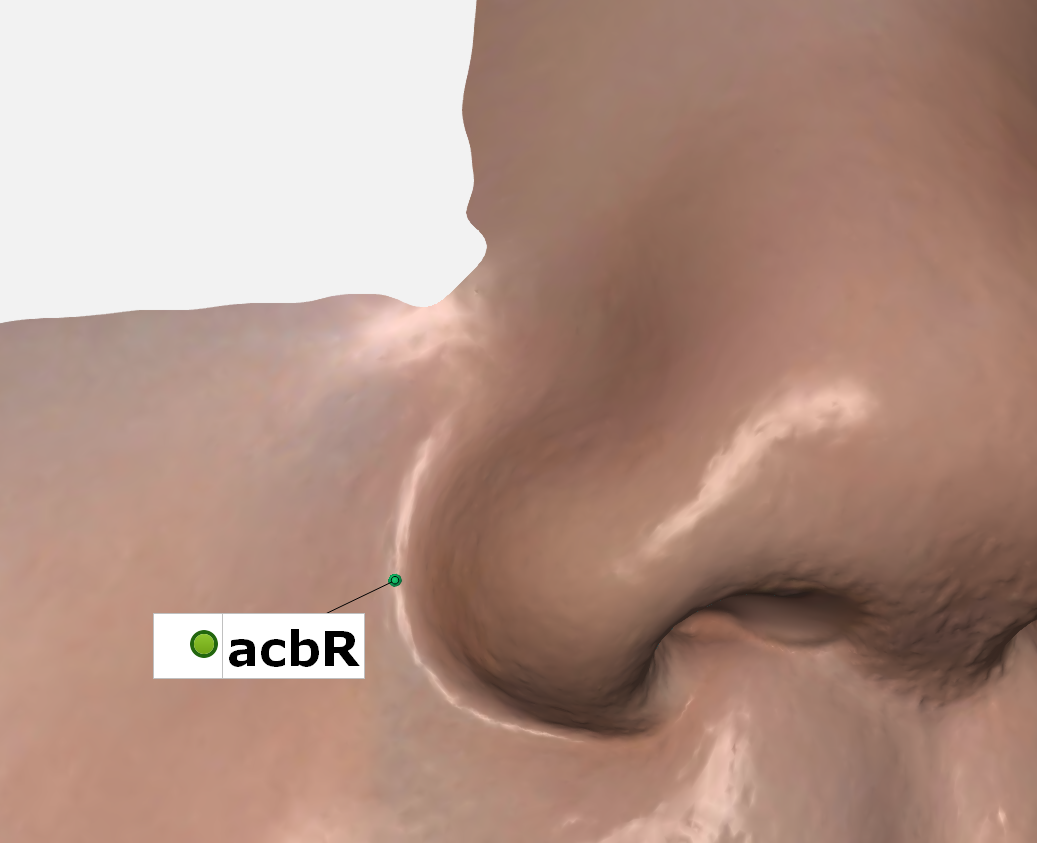 |
| Alare (alR, alL; 2:46) | Most lateral point on each alar contour. 3D anthropometrically, the landmarks are set in the frontal view after alignment in the morphological median sagittal. Identical to acbR/acbL in most cases in infants, not in adults.  *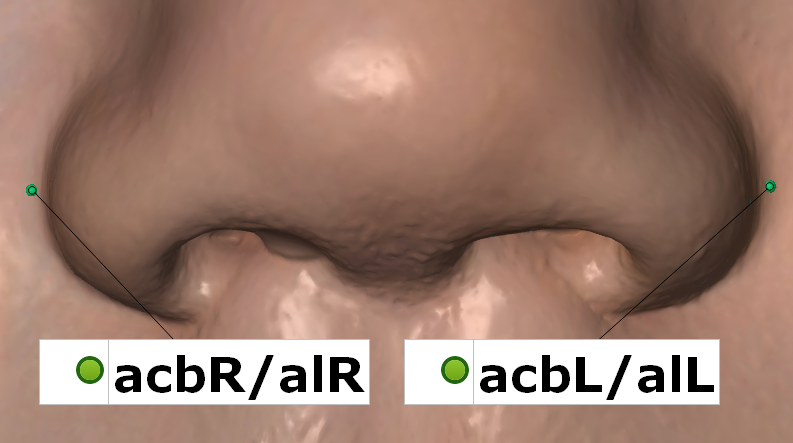* |
| High points of columella (cR, cL, 3:25) | The point on each columella crest level with the top of the corresponding nostril. 3D anthropometrically, the landmarks are set in the caudal view.  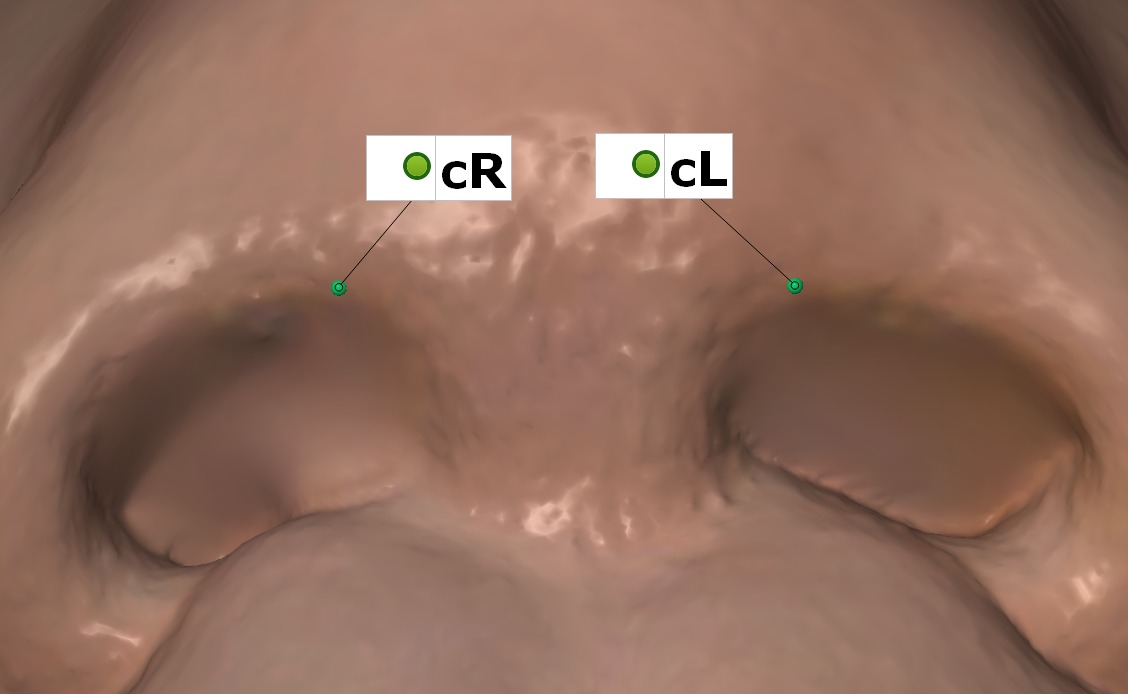 |
| Columellar median high point (cM 4:02) | Highest morphological median sagittal point on the columellar crest. 3D anthropometrically, the point can be identified at the intersection of the surface curves between cR and cL and between pr and sn.  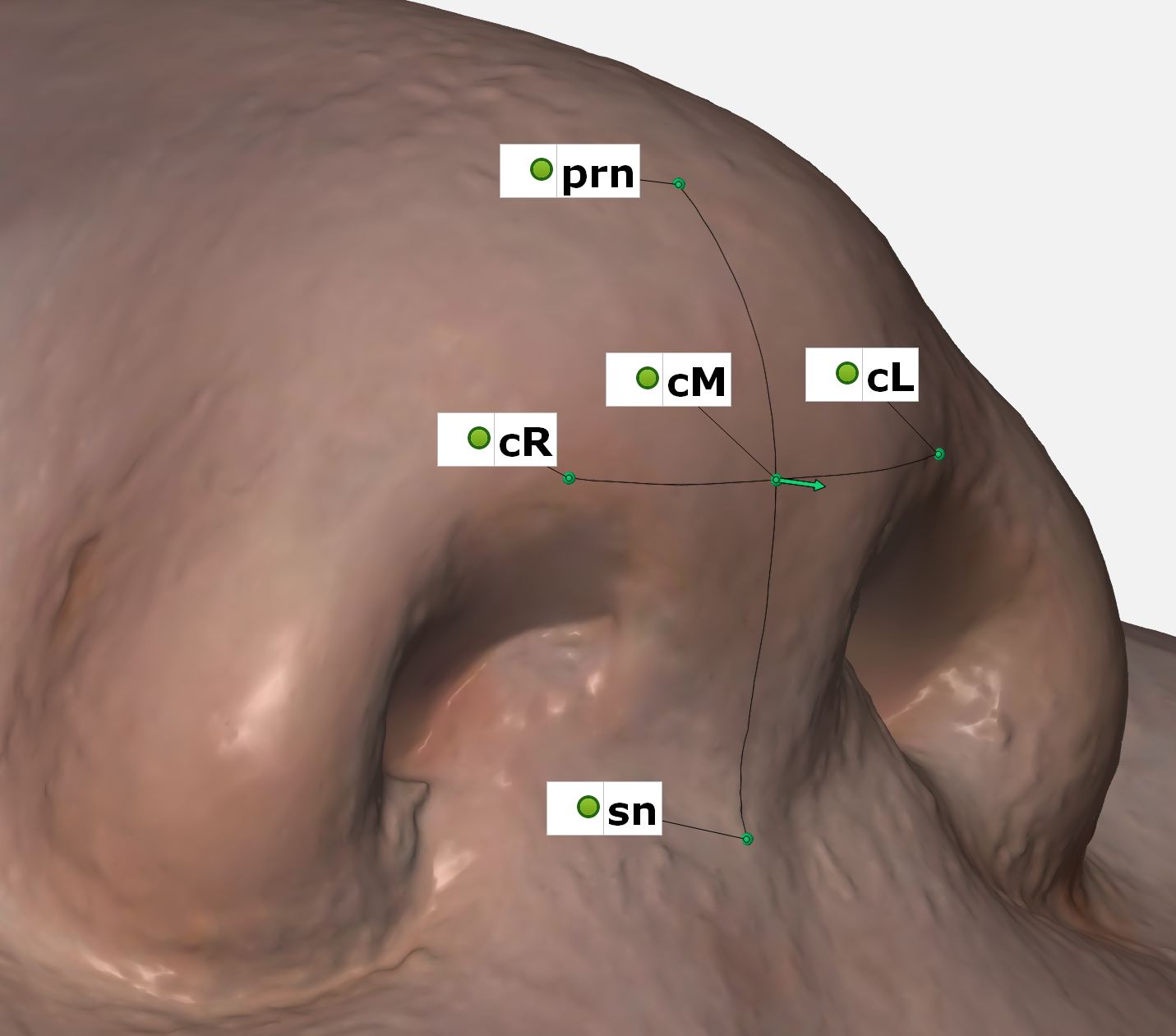 |
| Inner alare (aliR, aliL, 4:56) | Inner marking level at the most concentric midportion of the alae where the thickness of each ala is measured. The landmarks are situated at half-distance between the two attachment points of the alae, the anterior attachment point on the nasal body and the dorsal attachment point on the upper lip, respectively. 3D anthropometrically, the mesh should be positioned in caudal view to ensure correct positioning of the landmarks.  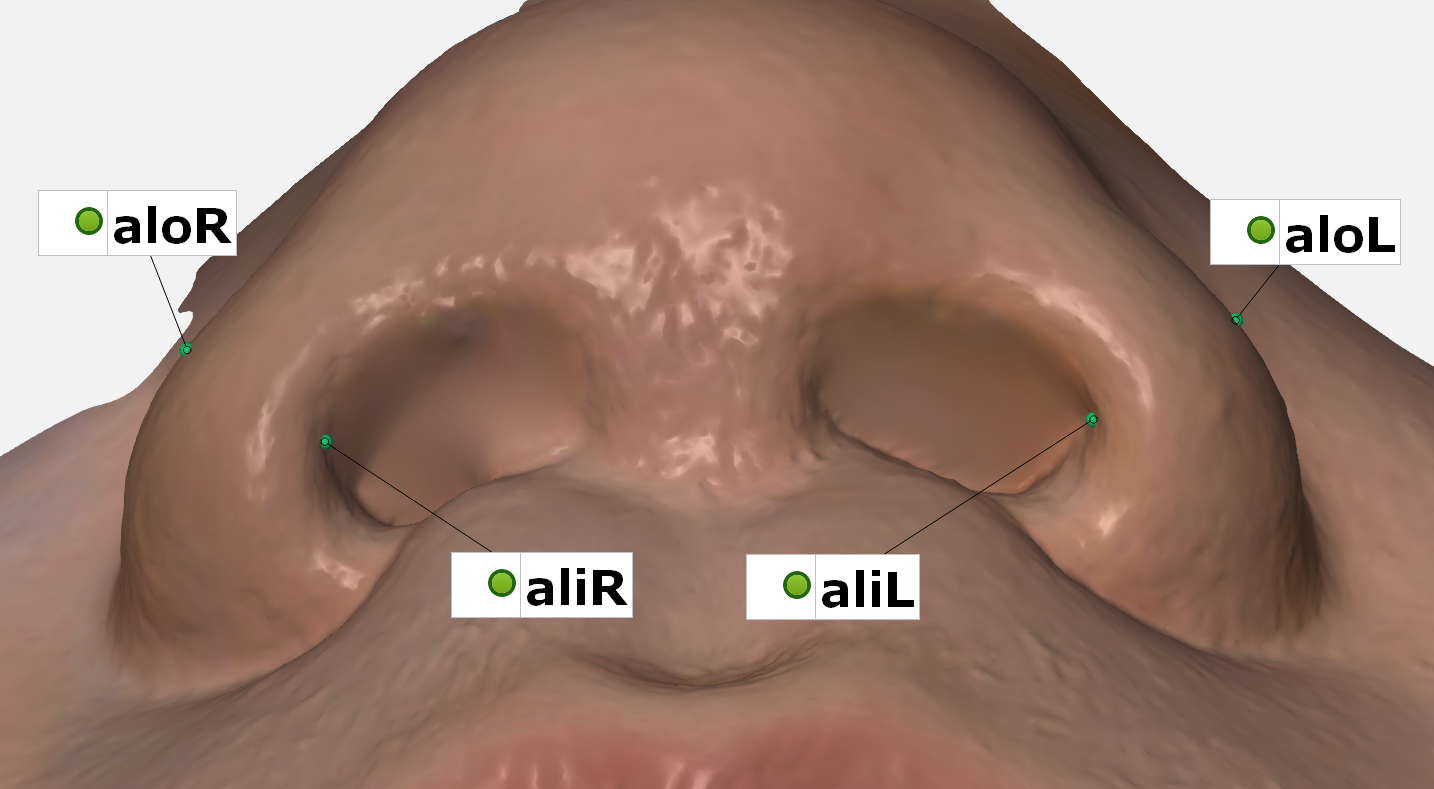 |
| Outer alare (aloR, aloL, 4:56) | Outer marking level at the most eccentric midportion of the alae where the thickness of each ala is measured. The landmarks are situated at half-distance between the two attachment points of the alae, the anterior attachment point on the nasal body and the dorsal attachment point on the upper lip, respectively. 3D anthropometrically, the mesh should be positioned in caudal view to ensure correct positioning of the landmarks. |
| Subalare (sbalR, sbalL, 5:28) | Point at the lower limit of each alar base where it joins the skin of the upper lip and touches an imaginary tangent at the caudal base of the nose. 3D anthropometrically, the landmarks can be identified by the light reflection that moves along the alarm base when the mesh is rotated around a longitudinal axis.  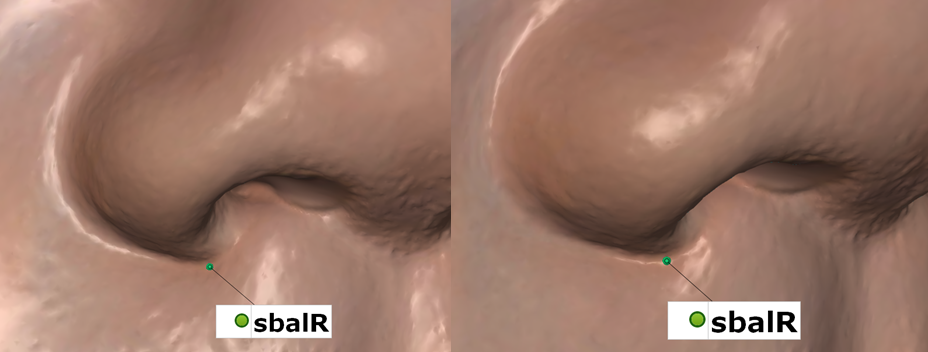 |
| Medial alar base (mabR, mabL, 6:03) | Point at the medial limit of each alar base where it meets the upper lip. 3D anthropometrically, the landmark is identified by following the light reflection that represents where the nose meets the facial/labial skin from the lateral to the most medial point. MabR/mabL is identical to cbR/cbL in alar base type 3.  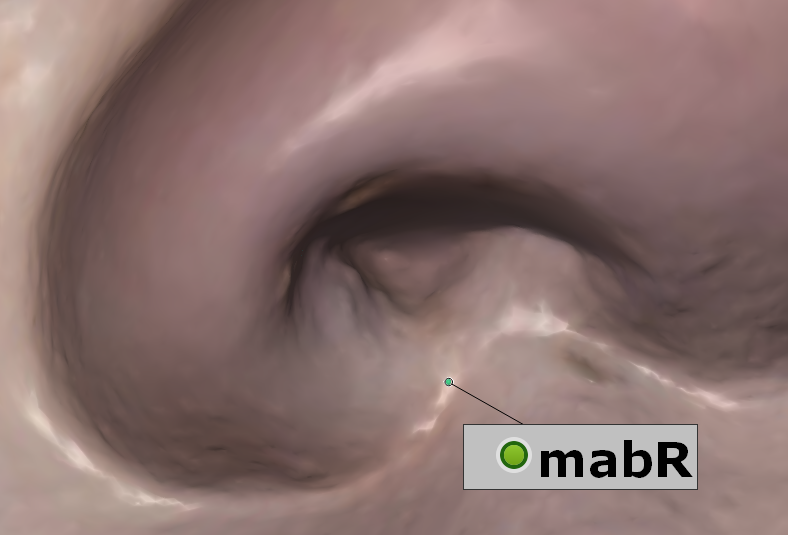 |
| Columella base (cbR, cbL, 6:40) | Most caudal and lateral point of the columella on each side at the bottom line where it meets the upper lip skin. 3D anthropometrically, the landmark is identified by following the light reflection that represents where the columella meets the labial skin from the medial to the most lateral point.  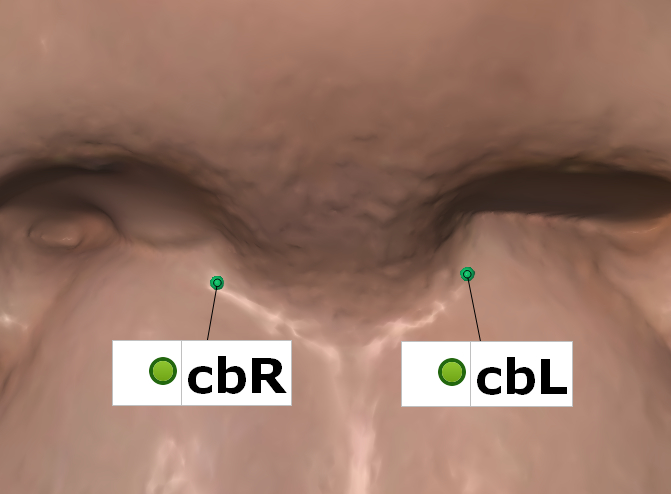 |
| Cheilion (chR,chL) | Point at each labial commissure. 3D anthropometrically, it may be helpful to follow the reflection and the difference in colour on the vermillion line or the reflection at the junction of the upper and lower lip to its most lateral point on each side. |
| Christa philtri (cphR, cphL) | Point on each elevated margin of the upper lip, at the junction of the vermillion line and the white roll, representing the cranial extremes of the Cupid's bow. 3D anthropometrically, the landmark is identified by the change in surface reflection on the vermillion to a continuous line on the vermillion edge when the mesh is rotated around a transversal axis in a slightly laterally angled view.   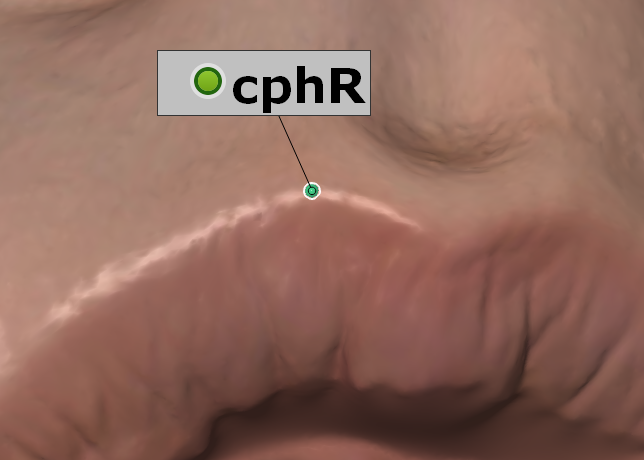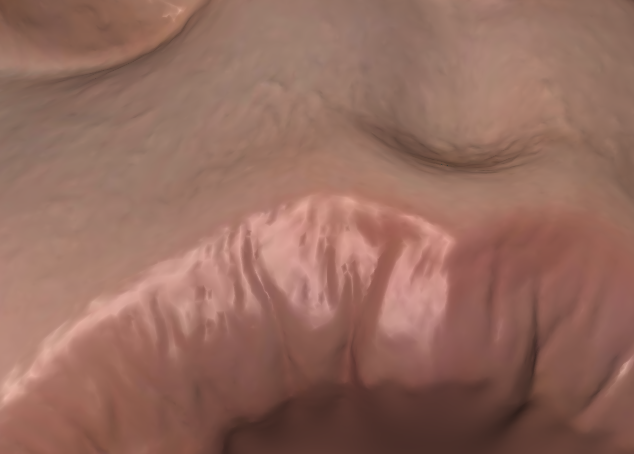 |
| Labrale superius (ls, 8:30) | Caudal midpoint of the upper lip, at the junction of the vermillion line and the white roll, representing the caudal extreme of the Cupid's bow. 3D anthropometrically, the landmark is identified at the junction of the previously identified reflection lines on the left and right vermillion edges. |
| Stomion (sto, 9:15) | Connection point between the upper and lower lip on the midline. 3D anthropometrically, it is defined as an imaginary point in the continuation of a vertical nasiolabial surface curve netween sn and ls (volumetric philtrum length) to the point where the upper lip touches the lower lip when slightly closed. In case of an open mouth scan, the landmark is created virtually at the estimated point of contact of the lips.  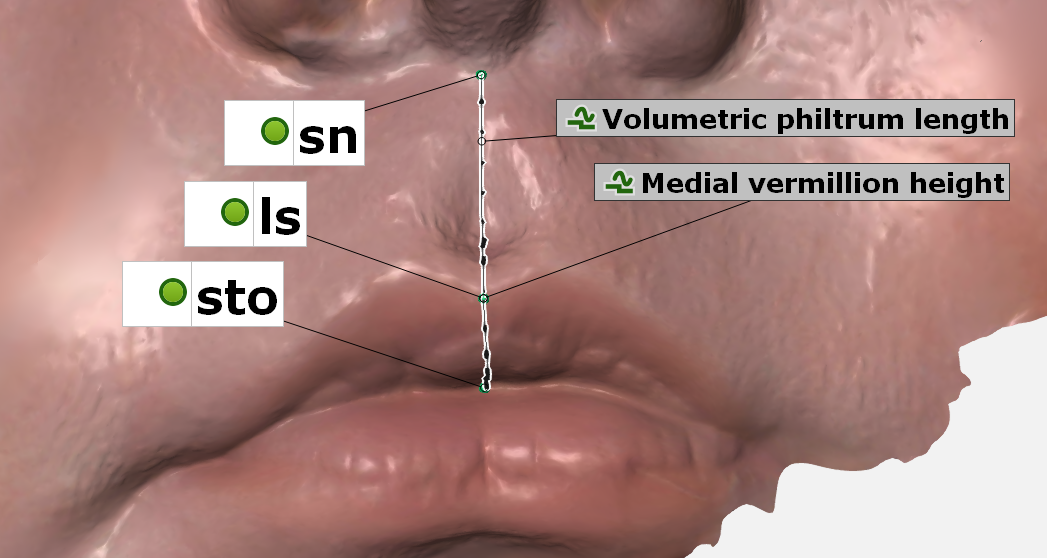 |
| Vermillion-mucosal junction (vmjR/vmjL/vmjM, 10:00) | Vermillion-mucosal junctions below the cranial and caudal extremes of the Cupid's bow. 3D anthropometrically, the landmark can be identified on a connecting surface curve between cphR/cphL/ls and stos at the point where the colour changes and the surface reflection simultaneously changes from inhomogeneous to homogeneous. This is best achieved by repeatedly rotating the mesh around the transverse axis.  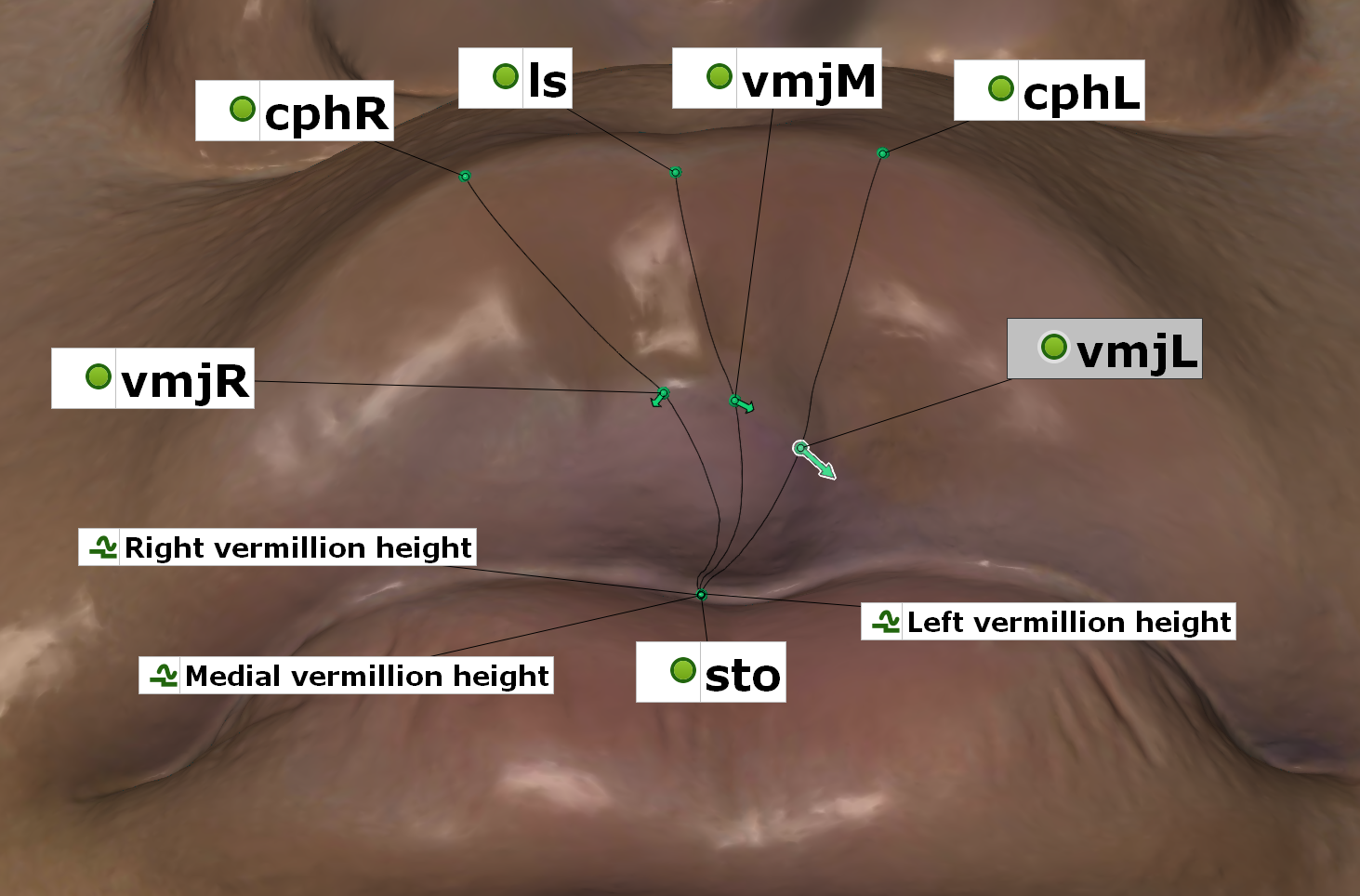 |
| Prolabiale (prl, 11:21) | Most protruded point on the upper lip in the morphological median sagittal. 3D anthropometrically, the landmark can be identified on the surface curve connecting sn and ls (volumetric philtrum length), with the distance between n and sn (nasal height) as the vertical reference plane, and chR and chL overlapping in the lateral view. AcP allows quantitative measurement of dimple depth.  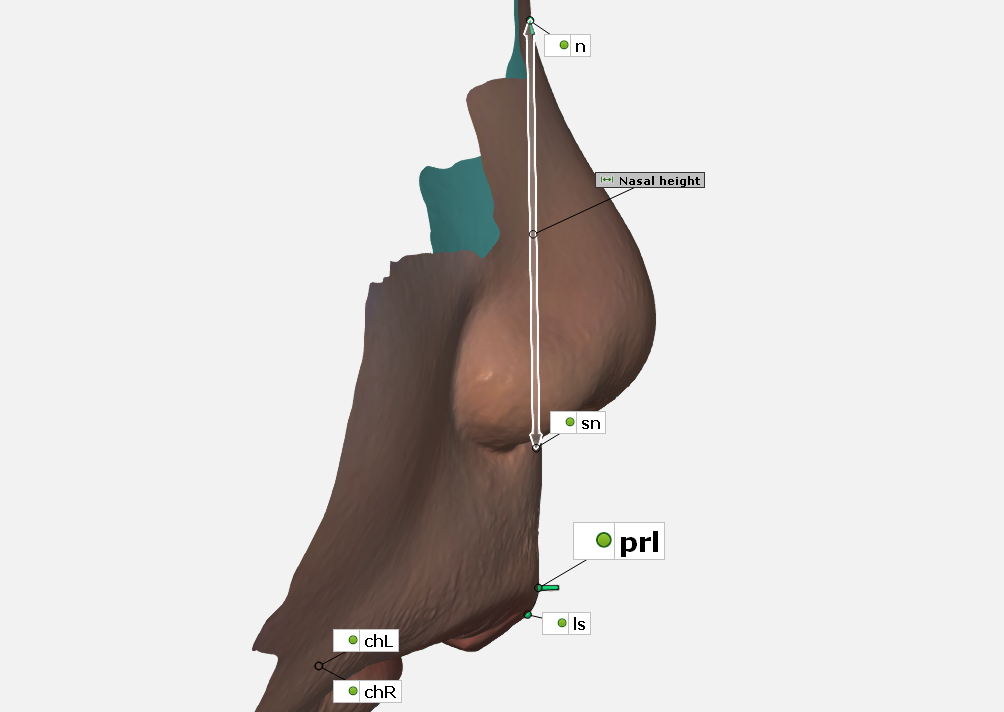 |
| Superior labial sulcus (sls, 12:24) | Deepest point of the superior labial sulcus (dimple). 3D anthropometrically, the landmark is identified as the point furthest from a direct line connecting sn and prl on the surface curve between sn and prl/ls.  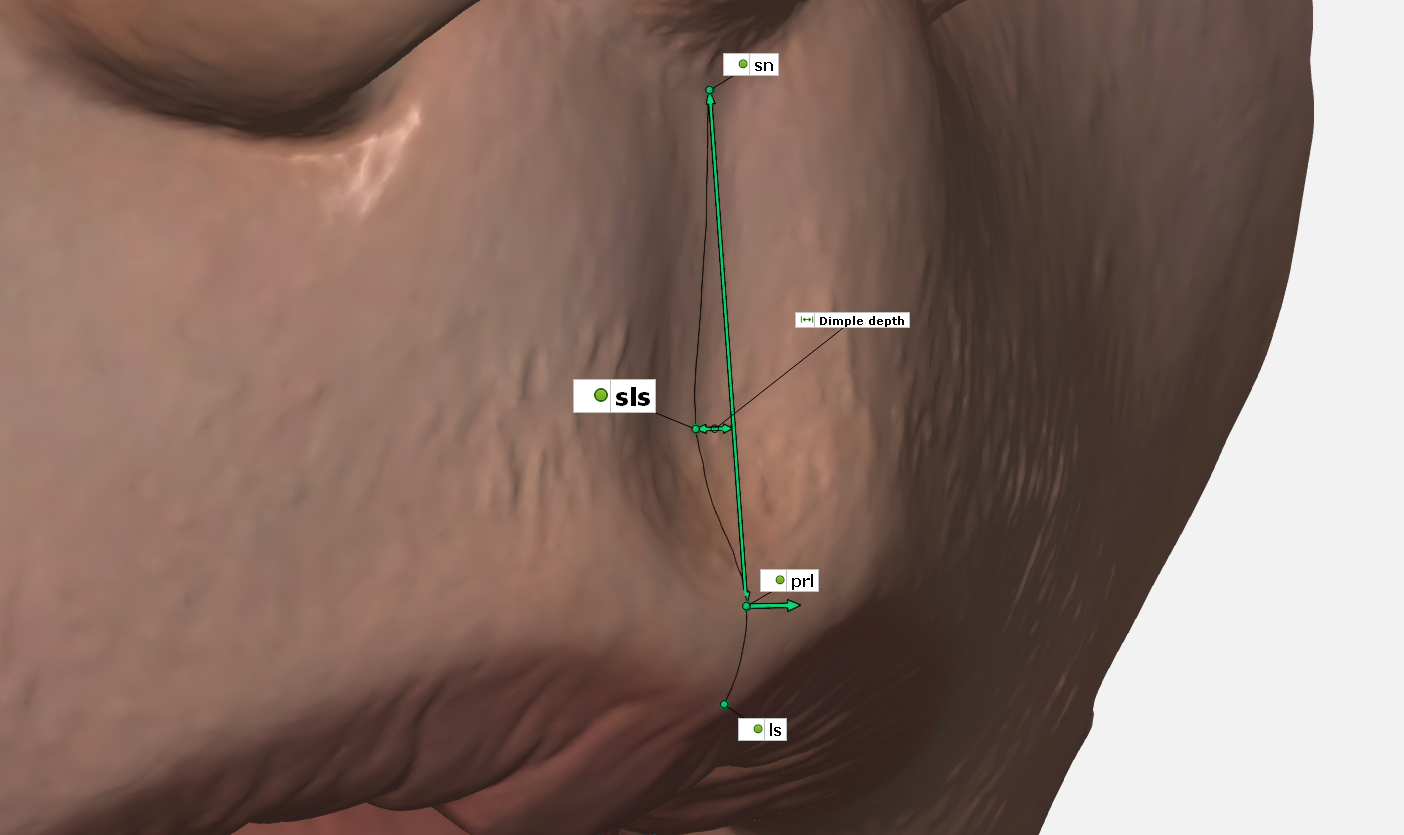 |
| Lateral vermillion (lvR,lvL, 13:17) | Most protruded point of the vermillion convexity. 3D anthropometrically, the landmark can be identified as the point furthest from a connecting line between chR/chL and ls (right and left vermillion length) on the vermillion edge reflection previously identified when setting cphR, cphL and ls.  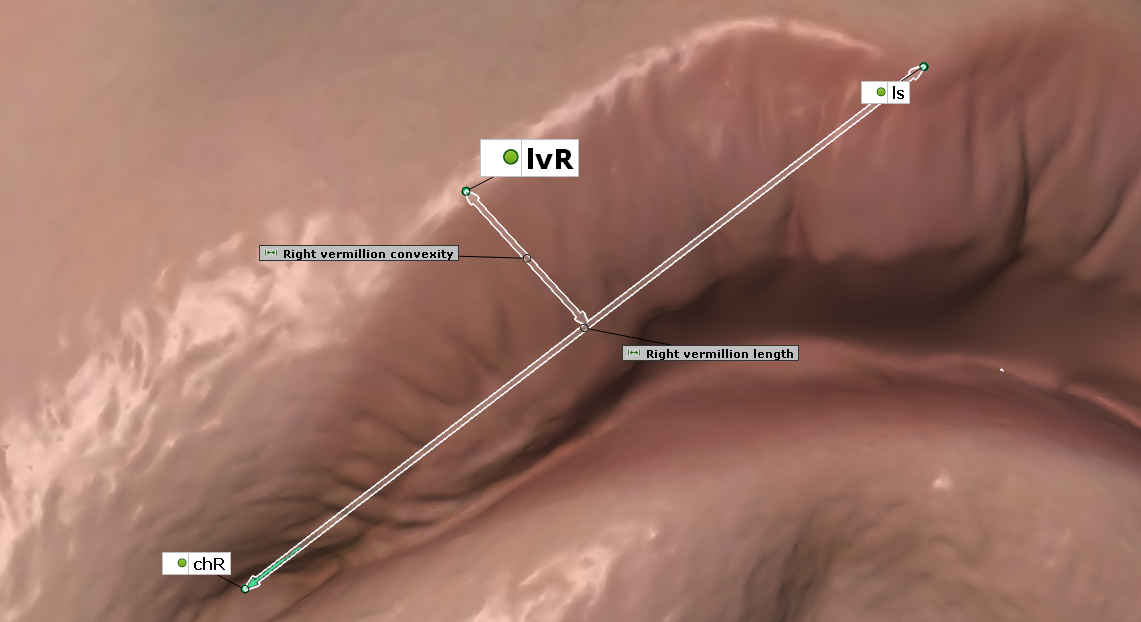 |

| **3D Measurements** | **Definition** |
| --- | --- |
| **Distances**  (corresponding number in Figure 1) | **Direct length between two landmarks or between a landmark and a point on a previous measurement** |
| Nasal height (D1) | n-sn |
| Nasal base width (D2) | acbR-acbL |
| Nasal alar width (D3) | alR-alL |
| Effective nasal length (D4) | Shortest distance (perpendicular length) between prn and a connecting line between n and sn (nasal height). |
| Right alar base to pronasale (D5) | acbR-prn |
| Left alar base to pronasale (D6) | acbL-prn |
| Right alar thickness (D7) | aloR-aliR |
| Left alar thickness (D8) | aloL-aliL |
| Caudal nasal base width (D9) | sbalR-sbalL |
| Right nostril floor width (D10) | mabR-cbR |
| Left nostril floor width (D11) | mabL-cbL |
| Right lateral columella length (D12) | cR-cbR |
| Left lateral columella length (D13) | cL-cbL |
| Median sagittal columella length (D14) | cM-sn |
| Mouth width (D15) | chR-chL |
| Dimple depth (D16) | Shortest distance (perpendicular length) between sls and a connecting line between acp and sn. |
| Vertical heights of the right lateral lip (D17) | sbalR-cphR |
| Vertical heights of the left lateral lip (D18) | sbalL-cphL |
| Vertical heights of the right medial lip (Philtrum) (D19) | cbR-cphR |
| Vertical heights of the left medial lip (Philtrum) (D20) | cbL-cphL |
| Upper lip height (D21) | sn-sto |
| Philtrum length (D22) | sn-ls |
| Right vermillion length (D23) | chR-ls |
| Left vermillion length (D24) | chL-ls |
| Transverse lengths of the right lateral lip element (D25) | chR-cphR |
| Transverse lengths of the left lateral lip element (D26) | chL-cphL |
| Right vermillion convexity (D27) | Shortest distance (perpendicular length) between lvR and a connecting line between chR and cphR (transverse lengths of the right lateral lip element). |
| Left vermillion convexity D28) | Shortest distance (perpendicular length) between lvL and a connecting line between chL and cphL (transverse lengths of the left lateral lip element). |
| Right lateral mouth to nose distance (D29) | chR-acbR |
| Left lateral mouth to nose distance (D30) | chL-acbL |
| Right median-sagittal mouth to nose distance (D31) | ls-cbR |
| Left median-sagittal mouth to nose distance (D32) | ls-cbL |
| **Surface curves**  (corresponding number in Figure 1) | **Curve between two landmarks positioned on the surface of the mesh (raw placement of surface curves without intersection point is indicated on the measurements with no further comments)** |
| Volumetric upper nasal height (S1) | n-prn |
| Volumetric lower nasal height (S2) | prn-sn |
| Volumetric nasal height (S3) | n-prn-sn |
| Volumetric soft-tissue nasal width right (S4) | acbR-prn |
| Volumetric soft-tissue nasal width left (S5) | prn-acbL |
| Right inner nostril circumference (S6) | Inner nostril circumference starting and ending at cbR; measured on the most concentric portion of the nostrils touching cbR, mabR and aliR. |
| Left inner nostril circumference (S7) | Inner nostril circumference starting and ending at cbL; measured on the most concentric portion of the nostrils touching cbL, mabL and aliL. |
| Right volumetric lateral columella length (S8) | Connecting cR-cbR on the columella crest; part of the right inner nostril circumference. |
| Left volumetric lateral columella length (S9) | Connecting cL-cbL on the columella crest; part of the left inner nostril circumference. |
| Right columellar base width (S10) | cbR-sn |
| Left columellar base width (S11) | sn-cbL |
| Columellar base width (S12) | cbR-sn-cbL |
| Volumetric philtrum length (S13) | sn-ls |
| Philtrum base width (Volumetric) (S14) | Connecting cphR-ls-cphL on the cranial vermillion borders light reflection. |
| Medial vermillion height (S15) | ls-vmjM-stos |
| Right vermillion height (S16) | cphR-vmjR-stos |
| Left vermillion height (S17) | cphL-vmjL-stos |
| Volumetric upper lip height (S18) | sn-ls-stos |
| Right dry vermillion height (S19) | cphR- vmjR |
| Median dry vermillion height (S20) | ls- vmjM |
| Left dry vermillion height (S21) | cphL- vmjL |
| Right wet vermillion height (S22) | vmjR-sto |
| Median wet vermillion height (S23) | vmjM-sto |
| Left wet vermillion height (S24) | vmjL-sto |
| Right volumetric vermillion length (S25) | chR-ls measured on the cranial vermillion borders light reflection touching cphR. |
| Left volumetric vermillion length (S26) | chL-ls measured on the cranial vermillion borders light reflection touching cphL. |
| Vertical heights of the right medial lip (Philtrum, Volumetric) (S27) | Connecting cbR-cphR along the philtrum edge |
| Vertical heights of the left medial lip (Philtrum, Volumetric) (S28) | Connecting cbL-cphL along the philtrum edge |
| Right philtrum base width (Volumetric) (S29) | Connecting cphR-ls on the cranial vermillion border. 3D anthropometrically, the surface curve is placed at the switch in light reflection when the mesh is rotated around an imaginary longitudinal axis. |
| Left philtrum base width (Volumetric) (S30) | Connecting ls-cphL on the cranial vermillion border. 3D anthropometrically, the surface curve is placed at the switch in light reflection when the mesh is rotated around an imaginary longitudinal axis. |
| Right lateral volumetric vermillion length (S31) | chR-cphR measured on the cranial vermillion borders light reflection. |
| Left lateral volumetric vermillion length (S32) | chL-cphL measured on the cranial vermillion borders light reflection. |
| **Angles**  (corresponding number in Figure 1) | **3D anthropometric angles as three-point angles or two-directional angles** |
| Columellar angle (A1) | Angle between cM-sn as first direction and the horizontal plane created by connection of the commissures chR-chL as second direction (two-directional angle) |
| Vermillion angle (A2) | chR-ls-chL with ls as angle point (three-point angle) |
| **Indices** |  |
| Vertical nasal convexity index | n-prn-sn / n-sn (surface curve div. by direct distance)  volumetric nasal height/nasal height |
| Alar convexity index | (acbR-prn + acbL-prn) / acbR-prn-acbL (surface curves div. by direct distance)  (volumetric soft-tissue nasal width right + volumetric soft-tissue nasal width left) / (right alar base to pronasale + left alar base to pronasale) |
| Right columella index | cR-cbR / cR-cbR (surface curve div. by direct distance)  right volumetric lateral columella length / right lateral columella length |
| Left columella index | cL-cbL / cL-cbL (surface curve div. by direct distance)  left volumetric lateral columella length / left lateral columella length |
| Columella index | (cR-cbR + cL-cbL) / (cR-cbR + cL-cbL) (surface curves div. by direct distance)  (right volumetric lateral columella length + left volumetric lateral columella length) / (right lateral columella length + left lateral columella length) |
| Horizontal nasal convexity index | (acbR-prn + prn-acbL) / acbR-acbL (surface curves div. by direct distance)  (volumetric soft-tissue nasal width right + volumetric soft-tissue nasal width left) / nasal base width |
| Nasal convexity index | VNCI + HNCI / 2  (vertical nasal convexity index + Horizontal nasal convexity index) / 2 |
| Columella base symetry index | cbR-sn / sn-cbL (surface curves div. by surface curve)  right columellar base width / left columellar base width |
| Dimple index | sn-ls / sn-ls (surface curve div. by direct distance)  volumetric philtrum length / philtrum length |
| Philtrum taper index | cphR-ls-cphL / cbR-sn-cbL (surface curves div. by surface curve)  philtrum base width (Volumetric) / (right columellar base width + left columellar base width) |
| Upper lip index | sn-ls-stos / sn-stos (surface curve div. by direct distance)  volumetric upper lip height / upper lip height |
| Right vermillion index | chR-ls / chR-ls (surface curve div. by direct distance)  right volumetric vermillion length / right vermillion length |
| Left vermillion index | chL-ls / chl-ls (surface curve div. by direct distance)  left volumetric vermillion length / left vermillion length |
| Vermillion index | (chR-ls + chL-ls) / (chR-ls + chL-ls) (surface curves div. by direct distance)  (right volumetric vermillion length + left volumetric vermillion length) / (right vermillion length + left vermillion length) |
| Lateral mouth to nose symetry index | (chR-acbR + cphR-sbalR) / (chL-acbL + cphL-sbalL) (direct distances div. by direct distances)  (right lateral mouth to nose distance + vertical heights of the right lateral lip) / (left lateral mouth to nose distance + vertical heights of the left lateral lip) |
| Central mouth to nose symetry index | (cphR-cbR + ls-cbR) / (cphL-cbL + ls-cbL) (direct distances div. by direct distances)  (vertical heights of the right medial lip (Philtrum) + right median-sagittal mouth to nose distance) / (vertical heights of the left medial lip (Philtrum) + left median-sagittal mouth to nose distance) |
| Mouth to nose symetry index | (LMTNSI + CMTNSI) / 2  lateral mouth to nose symetry index + central mouth to nose symetry index / 2 |
| Soft-tissue mouth to nose index | chR-chL / (alR or acbR-alL or acbL depending on which is the most lateral point) (direct distances div. by direct distances) |
