# Supplementary material for: 3D anthropometry of the nasolabial region in children aged 3 to 9 months as reference database for clinical assessment
Source: Sci Rep. 2025 Jul 28;15:27443. doi: 10.1038/s41598-025-11024-8 (PMC12304160; doi:10.1038/s41598-025-11024-8)
Supplement: Supplementary file 2 — Supplementary Material 2 [file 41598_2025_11024_MOESM2_ESM.pptx]

## Slide 1
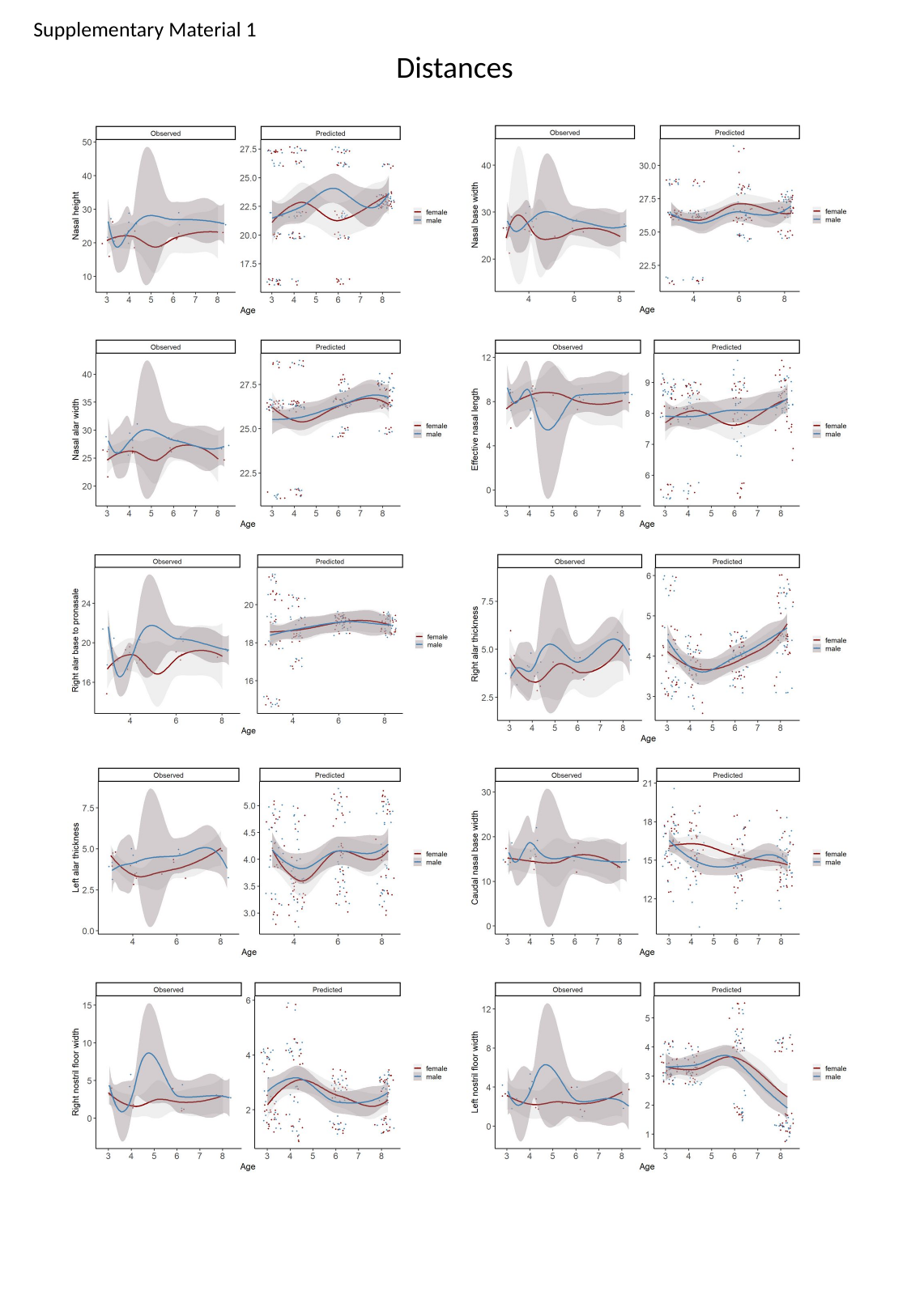

Figure 1
Supplementary Material 1
Distances

## Slide 2
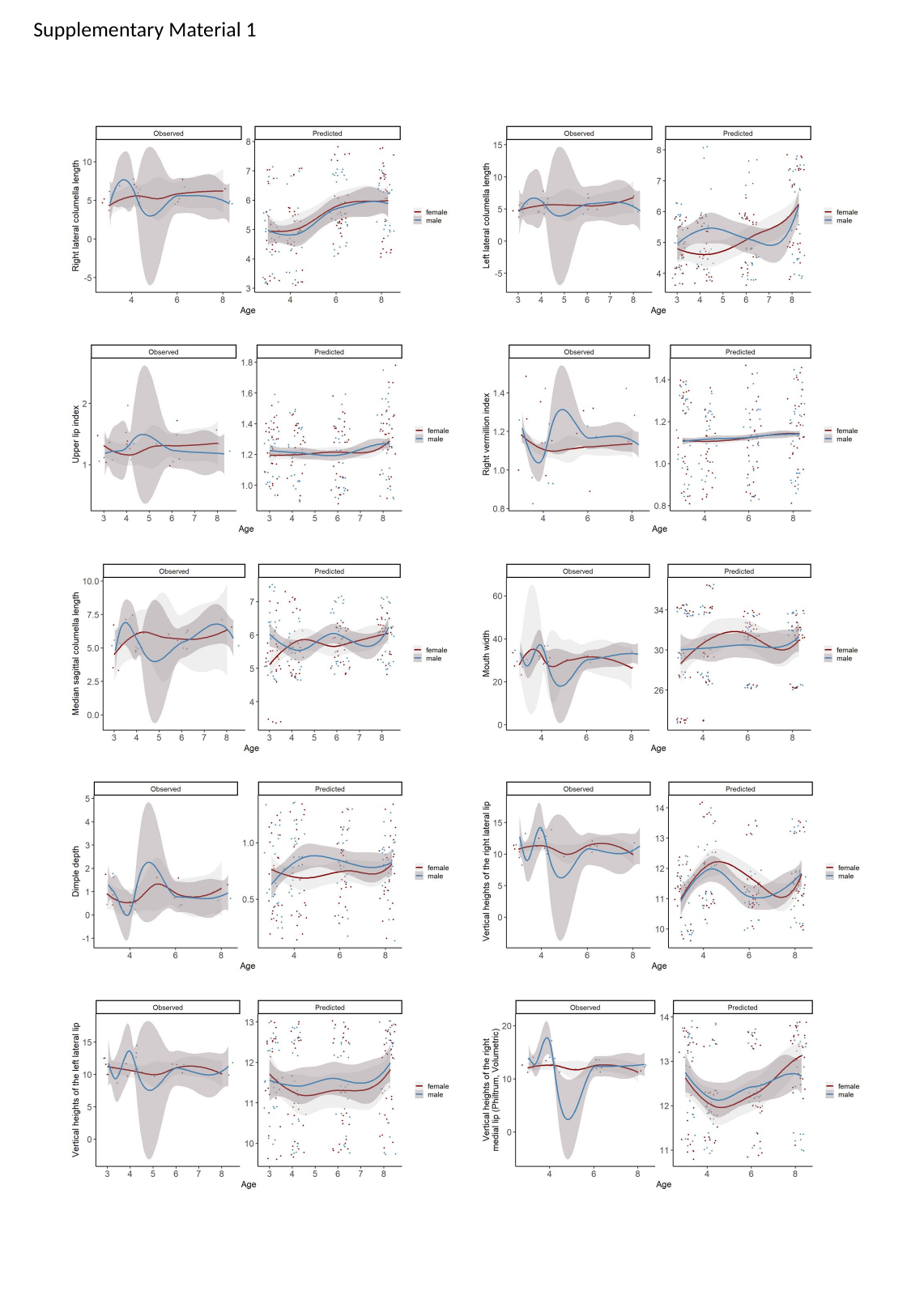

Figure 1
Supplementary Material 1

## Slide 3
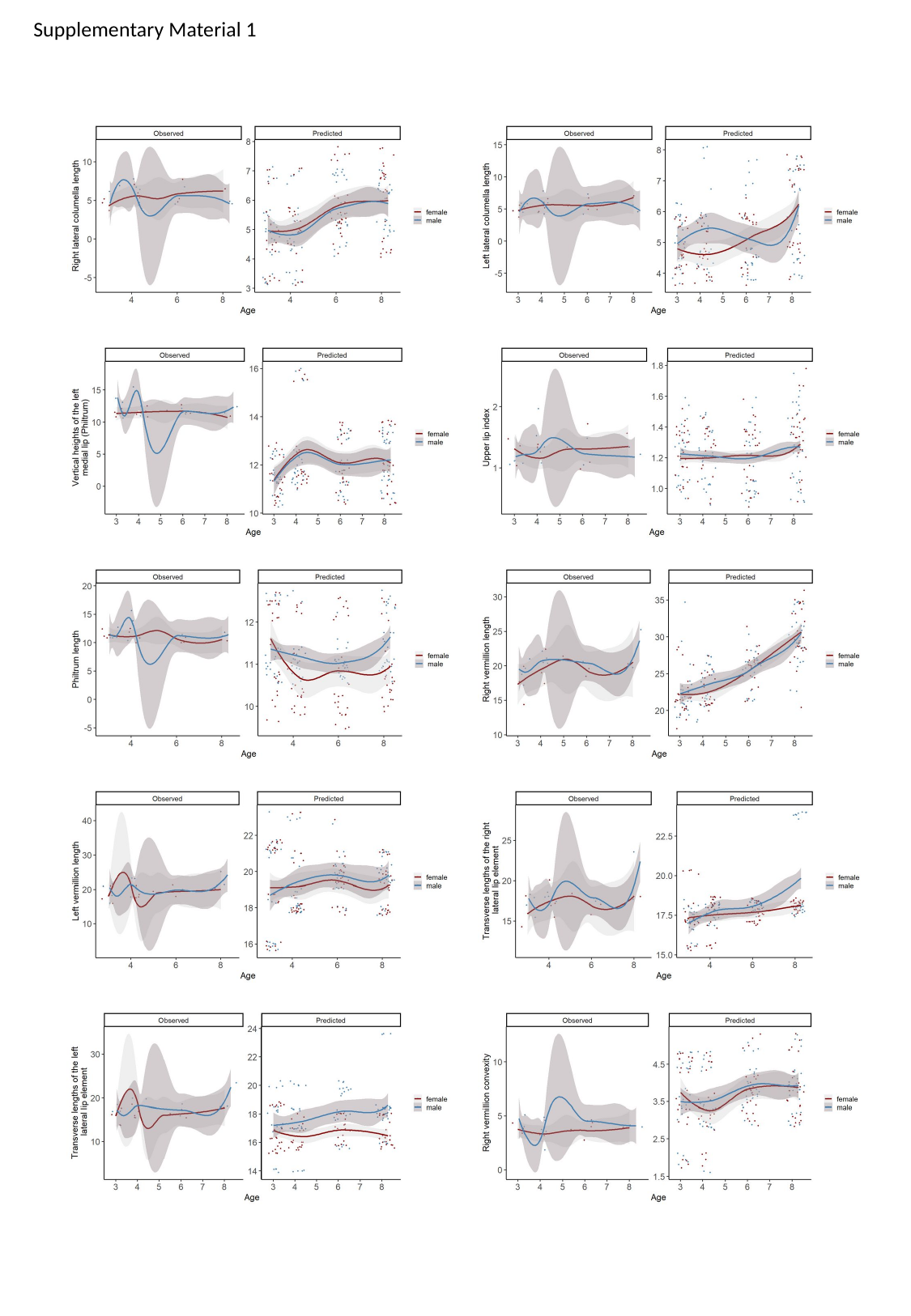

Figure 1
Supplementary Material 1

## Slide 4
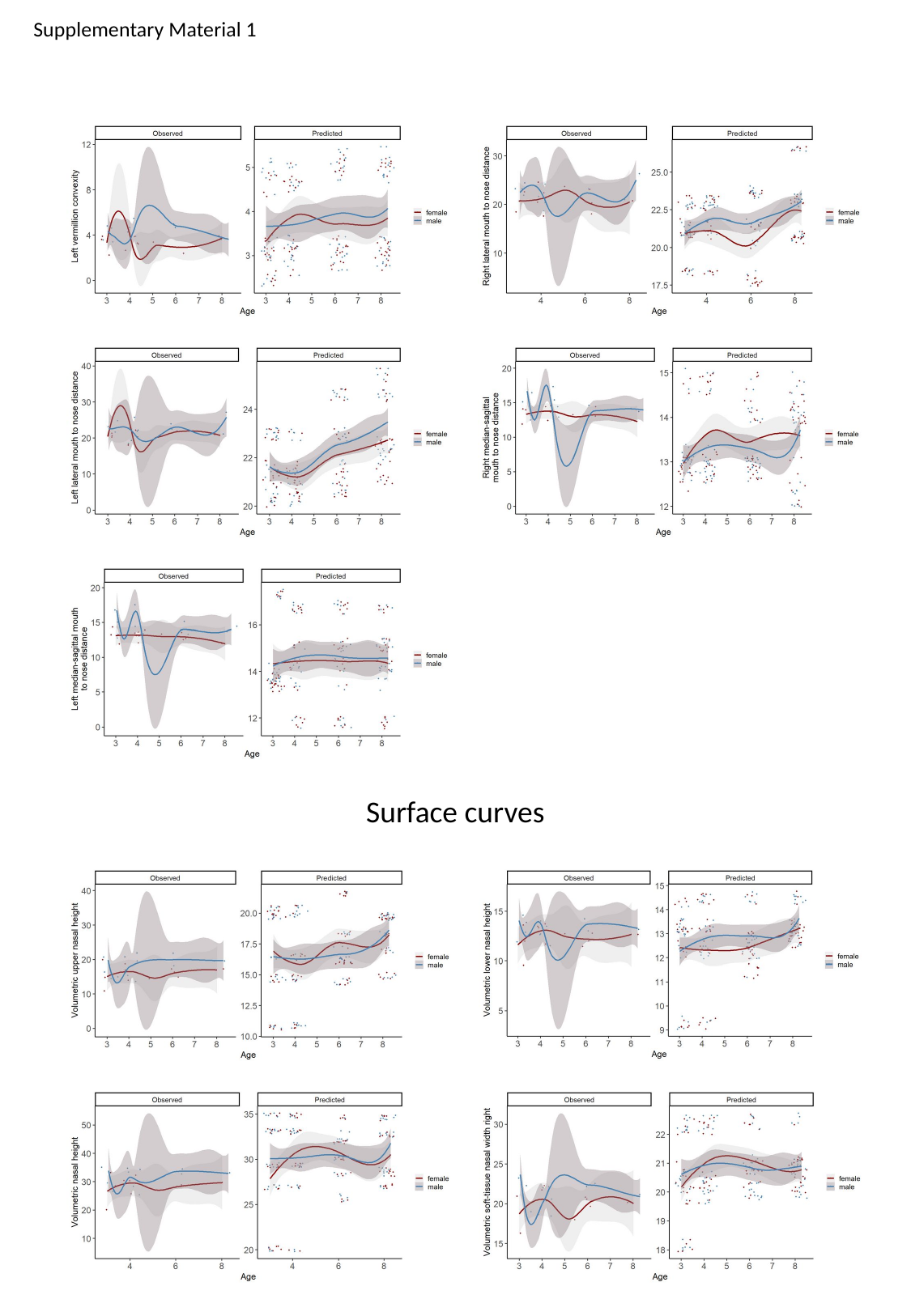

Figure 1
Supplementary Material 1
Surface curves

## Slide 5
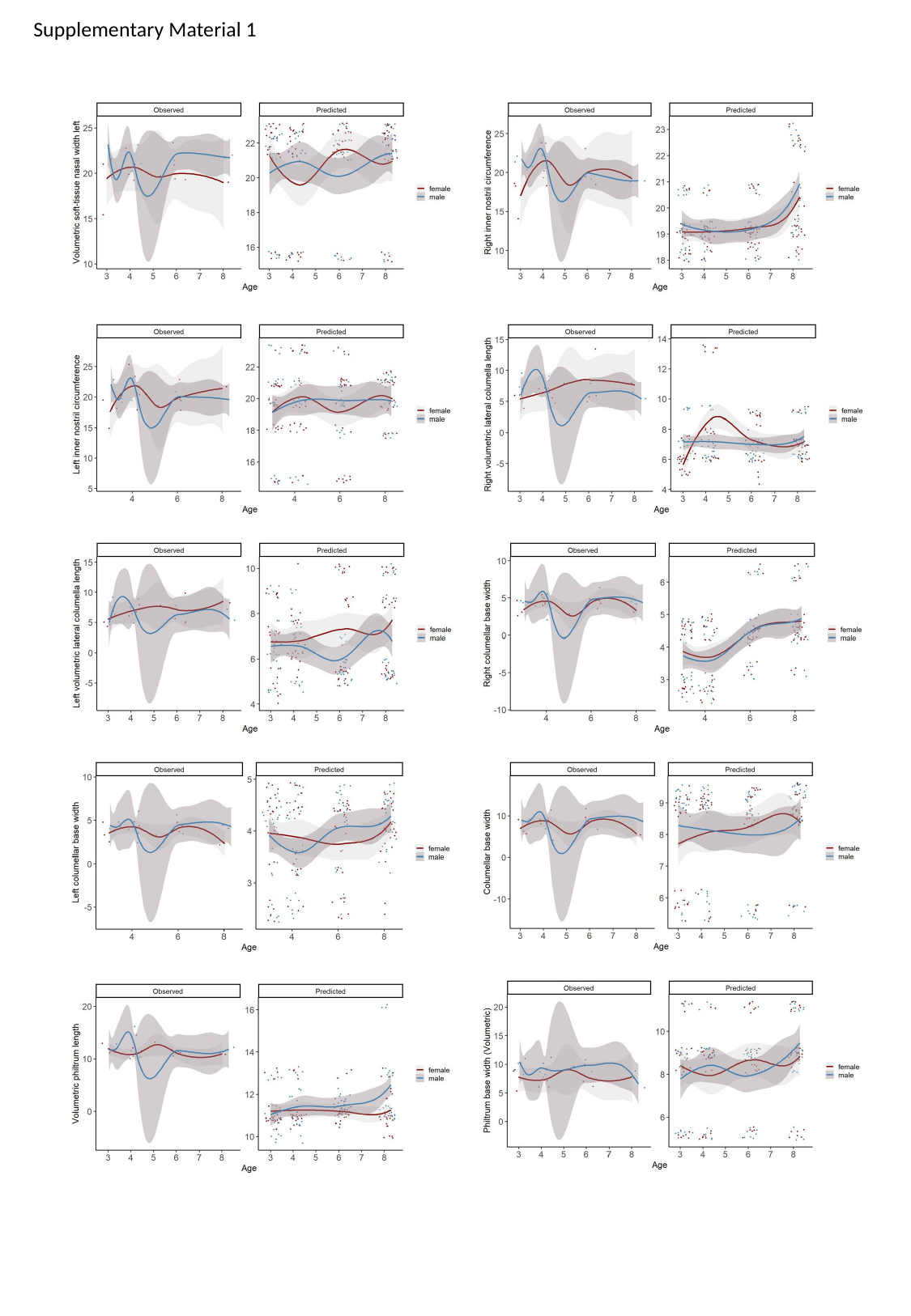

Figure 1
Supplementary Material 1

## Slide 6
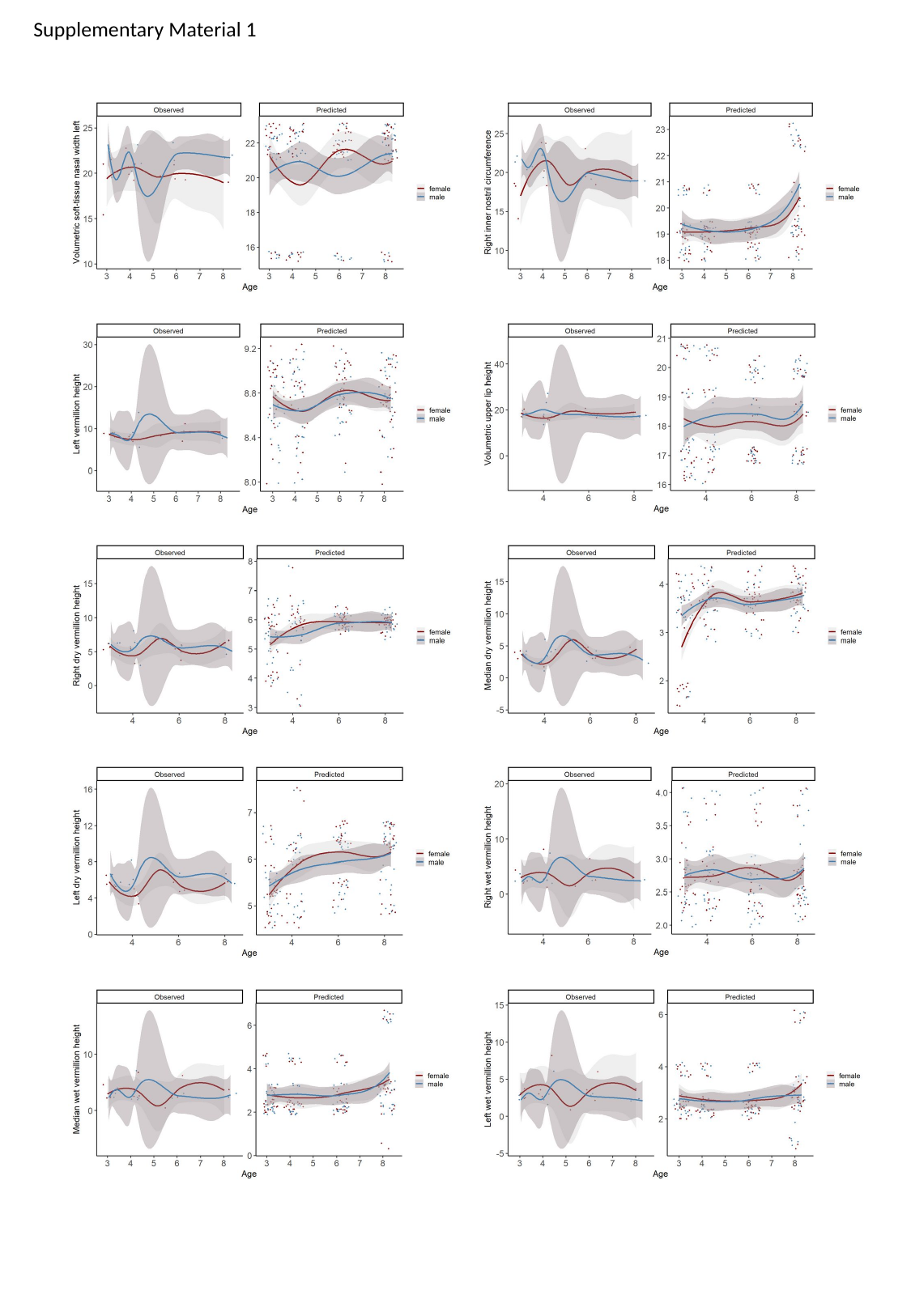

Figure 1
Supplementary Material 1

## Slide 7
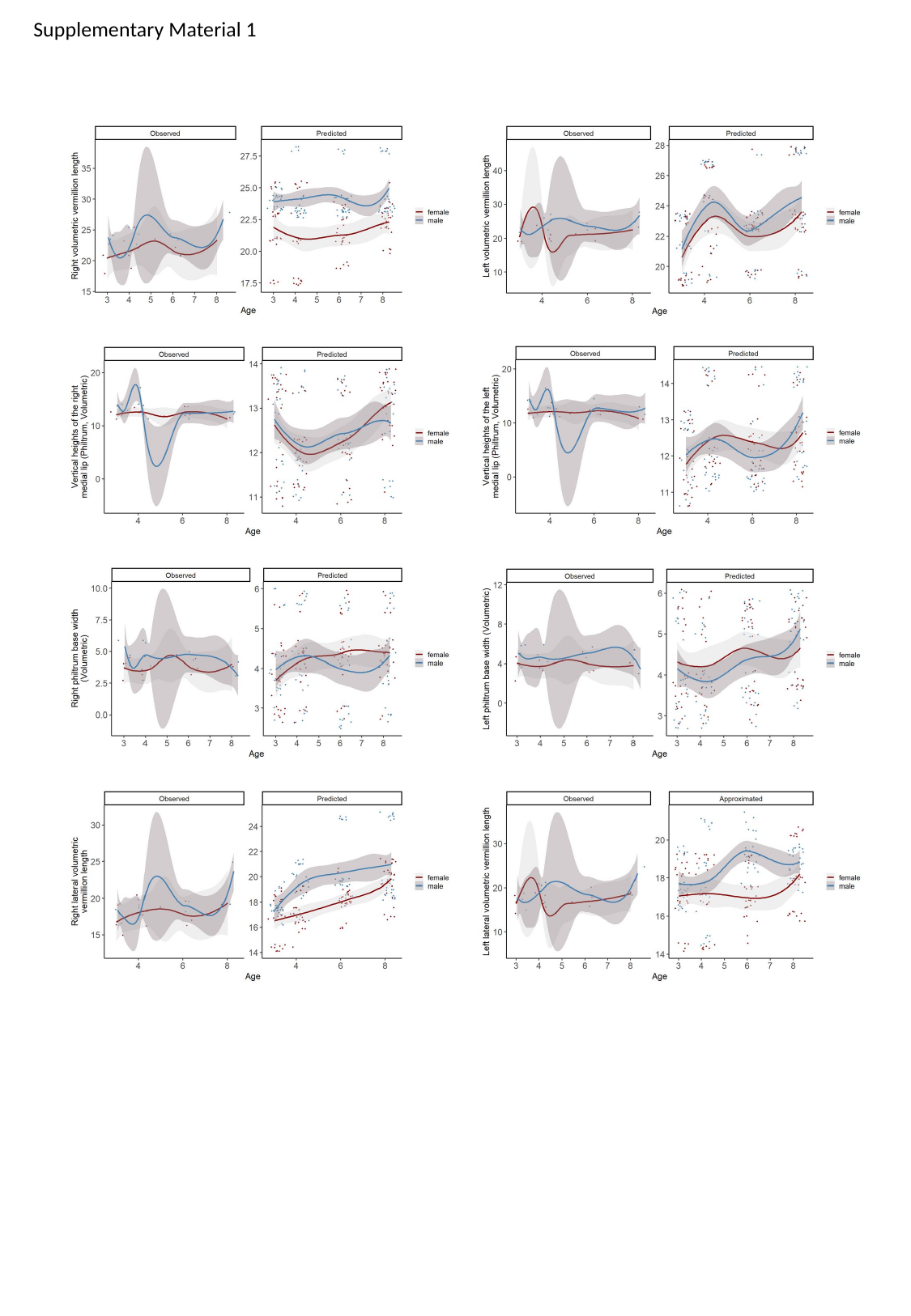

Figure 1
Supplementary Material 1

## Slide 8
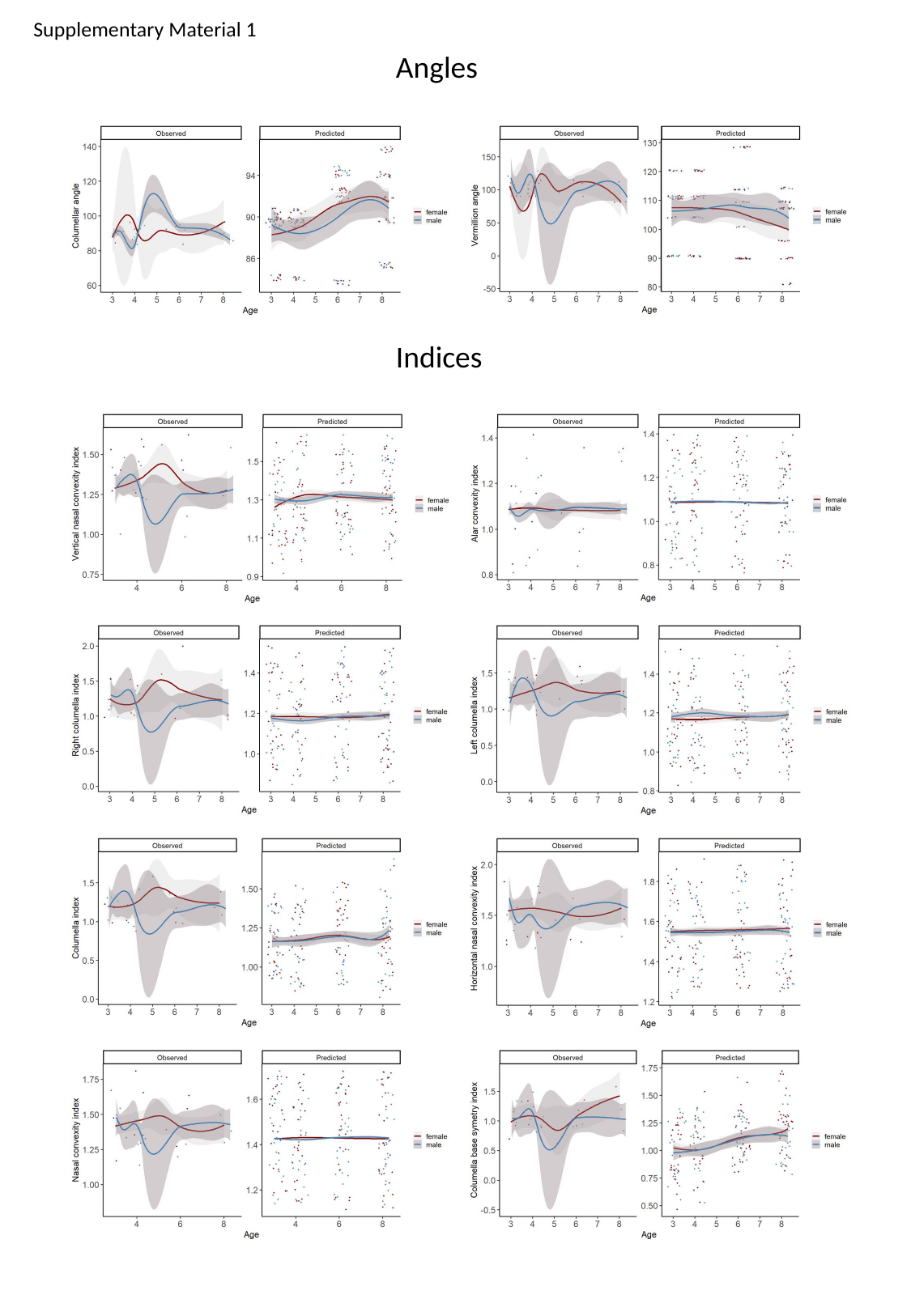

Figure 1
Supplementary Material 1
Angles
Indices

## Slide 9
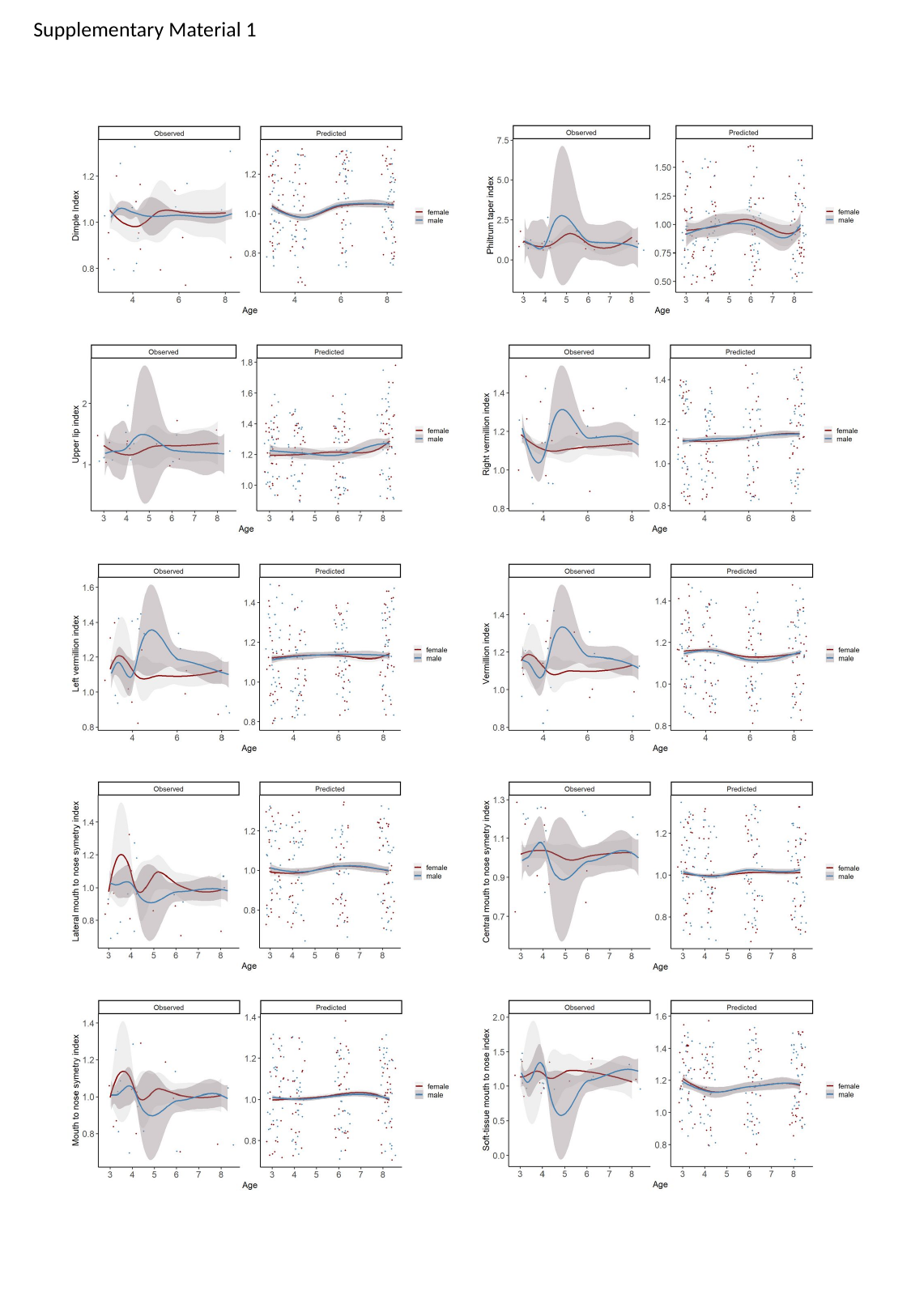

Figure 1
Supplementary Material 1
