# Supplementary material for: 3D anthropometry of the nasolabial region in children aged 3 to 9 months as reference database for clinical assessment
Source: Sci Rep. 2025 Jul 28;15:27443. doi: 10.1038/s41598-025-11024-8 (PMC12304160; doi:10.1038/s41598-025-11024-8)
Supplement: Supplementary file 5 — Supplementary Material 5 [file 41598_2025_11024_MOESM5_ESM.docx]

**Supplementary Material legend:**

**Supplementary Material 1**

**Overview and definition of the 33 3D anthropometric landmarks of the nasolabial region. Step-by-step tabular guide.**

This table is enhanced with images and supplements the concise version in the manuscript. To ensure precise and reproducible placement of the landmarks, Supplementary Material 4 provides an additional video guide.

**Supplementary Material 2**

**Descriptive plots of all 84 datasets for the 3D anthropometric parameters**

Descriptive plots for the 32 distances (Figure 1b), 32 surface curves (Figure 1c), 2 angles (Figure 1d) and the resulting 18 indices are listed. X-axis: age in months. Y-axis: length in millimetres, angle in degrees, and value of the indices in dimensionless numbers.

**Supplementary Material 3**

**Comprehensive mathematical equation for each parameter**

Equations are presented for both male and female patients, alongside their respective observed and predicted values. To determine the optimal morphological result for a particular parameter, it is necessary to enter the current clinical value as age in months in the equation. The authors recommend utilising the predicted value equations.

**Supplementary Material 4**

**Video guide for 3D anthropometric landmark positioning**

A short video guide for accurate and reproducible placement of the 33 3D anthropometric landmarks.
